# A Survey of Innovation through Duplication in the Reduced Genomes of Twelve Parasites

**DOI:** 10.1371/journal.pone.0099213

**Published:** 2014-06-11

**Authors:** Jeremy D. DeBarry, Jessica C. Kissinger

**Affiliations:** 1 Center for Tropical and Emerging Global Diseases, University of Georgia, Athens, Georgia, United States of America; 2 Department of Genetics, University of Georgia, Athens, Georgia, United States of America; 3 Institute of Bioinformatics, University of Georgia, Athens, Georgia, United States of America; Washington State University, United States of America

## Abstract

We characterize the prevalence, distribution, divergence, and putative functions of detectable two-copy paralogs and segmental duplications in the Apicomplexa, a phylum of parasitic protists. Apicomplexans are mostly obligate intracellular parasites responsible for human and animal diseases (e.g. malaria and toxoplasmosis). Gene loss is a major force in the phylum. Genomes are small and protein-encoding gene repertoires are reduced. Despite this genomic streamlining, duplications and gene family amplifications are present. The potential for innovation introduced by duplications is of particular interest. We compared genomes of twelve apicomplexans across four lineages and used orthology and genome cartography to map distributions of duplications against genome architectures. Segmental duplications appear limited to five species. Where present, they correspond to regions enriched for multi-copy and species-specific genes, pointing toward roles in adaptation and innovation. We found a phylum-wide association of duplications with dynamic chromosome regions and syntenic breakpoints. Trends in the distribution of duplicated genes indicate that recent, species-specific duplicates are often tandem while most others have been dispersed by genome rearrangements. These trends show a relationship between genome architecture and gene duplication. Functional analysis reveals: proteases, which are vital to a parasitic lifecycle, to be prominent in putative recent duplications; a pair of paralogous genes in *Toxoplasma gondii* previously shown to produce the rate-limiting step in dopamine synthesis in mammalian cells, a possible link to the modification of host behavior; and phylum-wide differences in expression and subcellular localization, indicative of modes of divergence. We have uncovered trends in multiple modes of duplicate divergence including sequence, intron content, expression, subcellular localization, and functions of putative recent duplicates that highlight the role of duplications in the continuum of forces that have shaped these genomes.

## Introduction

### Gene Duplications are Critical Components of Genome Evolution

What is the relationship between gene creation and organismal biology? Is genomic location an important factor in gene creation, maintenance, and potential for evolutionary innovation? While there are cases of apparent *de novo* gene creation, duplication of existing genes appears more common [1], [2]. Thus, to answer these questions it is necessary to first identify species-specific trends and patterns that link gene duplication, genome architecture, and organismal biology. Even a single gene duplication can have profound consequences. For example, Zimmerman *et al*. hypothesize that a single duplication of the *Plasmodium vivax* gene that encodes Duffy binding protein, which facilitates entry into red blood cells via binding to the Duffy blood group antigen, may be responsible for the increase of *P. vivax* human malaria observed in Duffy negative patients in sub-Saharan Africa [3].

Gene duplication mechanisms have been established (Reviewed in [4]–[7]), from studies of copy number variation and gene family amplifications [8]–[14], to the causes and effects of whole genome duplications [15], [16]. The innovative potential of paralogs can be explored via sequence, structure, and functional studies of the genes following duplication. Genome-scale data sets provide the means to discover the collective contribution of paralogs to gene repertoires, genome evolution, genome architecture, and adaptation across related species.

The actual contribution of duplications to genome evolution is sure to be greater than what can be detected. Much duplication is quickly removed, or has diverged beyond detection. Genome assemblies may underestimate duplications due to difficulties in assembling repetitive genomic regions, especially with short-read sequences. Despite these limitations, valuable information about the trends and patterns in gene creation and subsequent diversification (or not) can be gathered from the data that are available. Genomic distributions of duplications have been used to identify regions of rapid chromosomal evolution, where they presumably serve as catalysts for the generation of adaptive and diversifying functions [17], [18]. For example, duplications in the primate lineage have been implicated in gene creation, genome rearrangements, and potentially in shaping human genetic variation [19].

### Genome Cartography is an Effective Comparative Genomics Strategy

Genome sequences can serve as records of the adaptive histories of their evolution [20]. By contextualizing genomes as ancient fluid landscapes and mapping their features, it is possible to ‘get the lay of the land’, and to compare and contrast the evolutionary forces that have contributed to the variable genomic landscapes we observe [21]. Mapping the distributions of detected duplications can illustrate how they contribute to genome evolution and how they evolve with time. Thus, duplications can, and have, served as both a primary focus, and as tools to investigate molecular evolution within and between genomes [4], [6], [8], [18], [19]. In this study, we focus on an examination of duplications in the realm of intracellular parasites and extreme genome reduction.

### Apicomplexa: Characterization of Duplications in Twelve Species

**Figure 1:**
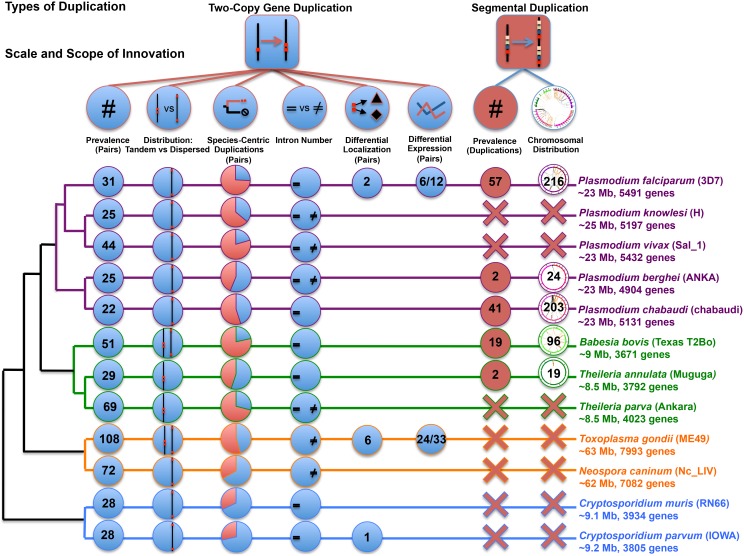
Scale, scope, and outcome of detected apicomplexan innovative duplication. Cladogram branch colors indicate major lineages. Strains follow species names. Genome sizes and protein-encoding gene counts are below each species name. Circles located on each branch contain counts, results, and trends for detected measures of innovation and are only present if data were available. For ‘Two-Copy Gene Duplication’, numbers are for total duplicate pairs or pairs with detected differences. ‘Species-Centric Duplications’ includes two categories: ‘species-specific pairs’ and ‘pairs with single copy ortholog’. ‘Differential Expression’ circles indicate detected differences over pairs with available data. A 60% cutoff was used to identify the major trend for ‘Distribution’ and ‘Intron Number’. Numbers in white circles are of unique genes in segmental duplications. White circles are scaled versions of distributions. Red ‘X’s = ‘none detected’.

**Figure 2:**
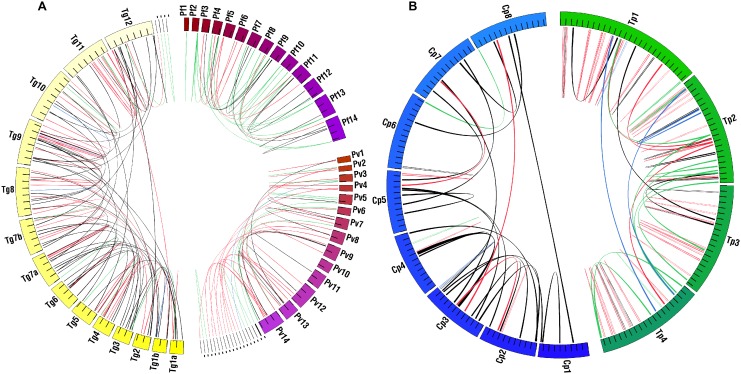
Duplicate gene distribution with respect to genome architecture. Colored circles represent the chromosomes and contigs in each genome (one color gradient/genome). Each species’ genome is labeled with the genus species abbreviation and chromosome/contig number Small unassembled contigs appear as black lines at the end of the respective genome sequence. Species are grouped based on genome size and karyotype. Tick marks = 1 Mb in **A** and 100 kb in **B**. Arcs connect two-copy paralog loci. All arcs have two ends; tandem copies may appear as a single line. Paralog start and stop coordinates on chromosomes/contigs are expanded for visualization. Arc colors identify ortholog copy number in the closest relative(s). Black = two-copy, Green = species-specific, Red = one copy, Blue>two-copy. Pf = *Plasmodium falciparum,* Pv = *P. vivax*, Tg = *Toxoplasma gondii,* Tp = *Theileria parva*, Cp = *Cryptosporidium parvum*. Red arrows in **B** indicate the fatty acid synthase and polyketide synthase genes.

The Apicomplexa are a eukaryotic phylum of unicellular parasites responsible for significant human and veterinary disease that affect millions worldwide. Diseases include malaria and AIDS-related toxoplasmosis and cryptosporidiosis. Only a single species has been shown to deviate from obligate intracellular parasitism [22]. Apicomplexans in this study are grouped into four lineages: *Plasmodium spp.* (agents of malaria), piroplasms (*Babesia* and *Theileria spp.*), coccidians (*Toxoplasma gondii* and *Neospora caninum)*, and *Cryptosporidium spp.* Genome sizes and chromosome numbers vary and gene repertoires are greatly reduced compared to model eukaryotes (Figure 1 and Figure 2). Relationships in Figure 1 are adapted from [21], [23]–[25]. Genomic streamlining and gene loss are major forces across the phylum (Reviewed in [21]). Given the relationship between gene repertoires and niche potential, the duplication of genetic material and its impact on host range and pathogenicity are of particular interest. Loss of synteny is very high compared to other eukaryotes, including other parasites [23], [26]. Transposable elements (TEs) which are ubiquitous in all other phyla studied to date, as well as major contributors to gene duplication and genome rearrangement, are conspicuously absent in most apicomplexans, including those studied here (Barrie A, Cheng S, Kissinger J, Pritham E, personal communication) [27], [28]. Apicomplexans offer an opportunity to explore the role of gene duplication in the evolution of parasite genome sequences over ∼420 my [29], [30], in the context of extreme genome reduction. Since apicomplexan genomes are streamlined, duplications detected in the phylum are likely important to parasite biology.

We used *in silico* methods to identify homologs in twelve species comprising four apicomplexan lineages and identify two-copy genes (duplicates) and segmental duplications in the genome assemblies and annotations of each species. We chose two-copy genes in an attempt to identify putative recent duplications and to design questions focused on chromosomal distribution and copy number evolution. We have investigated the scale, genomic distributions, general timing, divergence, and functional consequences of these duplications phylum-wide. For the Apicomplexa, these data represent the first step towards answering the questions posed above.

## Methods

### Data, Homolog Clusters, and Copy Number Determination

All data are from the most recent genome release at the time of analysis. Annotated protein-encoding gene sequences, gene IDs, functional annotations, gene coordinates, and the numbers, sizes, and IDs of all chromosomes/contigs/scaffolds for all species were obtained as follows: *P. falciparum, P. vivax, P. knowlesi, P. berghei,* and *P. chabaudi* data were downloaded from PlasmoDB [31] version 7.0. *C. muris* and *C. parvum* data were downloaded from CryptoDB [32] version 4.3. The 45 scaffolds from *C. muris* are not assigned to chromosomes and are numbered 1–45. *T. gondii* and *N. caninum* data were obtained from ToxoDB [33] version 6.2. *B. bovis, T. annulata, and T. parva* data were downloaded from NCBI (http://www.ncbi.nlm.nih.gov/) in March 2011. *B. bovis* data from chromosomes 1 and 4 were present in fragments (7 and 3 respectively) caused by genome assembly gaps. Accession numbers for these fragments are: Chromosome 1: AAXT01000005 AAXT01000006 AAXT01000008 AAXT01000009 AAXT01000010 AAXT01000011 and AAXT01000012, Chromosome 2: Nc_010574, Chromosome 3: Nc_0105745, Chromosome 4: AAXT01000002 AAXT01000004 and AAXT01000013.

Homologs were clustered using WU-BLAST (http://blast.wustl.edu/) (version 2.2.6), BLASTp [34], and OrthoMCL [35] as described in [23]. Two-copy paralogs were parsed from OrthoMCL output. As a guard against potential false positives, available annotation information was consulted when available. There were no cases of conflicting functional information among cluster members. Thirty-nine potential *P. falciparum* duplicate pairs were identified as completely overlapping gene models in PlasmoDB and removed. Twenty-three additional duplicate pairs were removed based on annotations from EuPathDB [36], either because they likely belong to larger gene families, or because they are annotated as pseudogenes. Ortholog copy numbers in the closest relative(s) were parsed from OrthoMCL output.

### Duplicate Gene Distributions

Gene coordinates were used to classify duplicates as dispersed or tandem. We define tandem duplicates based on the number of intervening genes, ≤5, and the distance between them, ≤1% total chromosome length. Most tandem duplicates have zero or one intervening genes. Some duplicates have one or both members on unassigned contigs. In these cases, the actual distribution could not be determined and these were counted as dispersed: *P. knowlesi* has three duplicate pairs where one member is on an unassigned contig, *P. vivax* has ten with both members unassigned and fifteen with one member unassigned, *P. chabaudi* has one with both members unassigned and two with one member unassigned, *P. berghei* has two with one member unassigned, *T. gondii* has two with both members unassigned and two with one member unassigned, *N. caninum* has one with both members unassigned and seven with one member unassigned.

### Segmental Duplications, Synteny, Gene Classes, and Visualization

Synteny and gene classes (core, species-specific, and multi-copy) were calculated as in [23]. Briefly, all homologs detected by OrthoMCL were input into MCSCAN [37] to calculate synteny between all combinations of genomes. Parameters for synteny detection are based on validated parameters from [23]. Gene classes were parsed from OrthoMCL output. MCSCAN returned segmental duplications as regions of within-genome synteny. Unique gene IDs for syntenic markers were extracted from MCSCAN output. The position of genes relative to chromosome ends and syntenic blocks are based on coordinates in MCSCAN output. Duplicates on unassigned contigs were not included in gap/end analyses. Visualizations of gene distributions, segmental duplications, and synteny were produced from gene coordinates and parsed MCSCAN output via Circos [38].

### Duplicate Diversification

Nucleotide sequences for *P. falciparum* and *T. gondii* duplicates were obtained from EuPathDB. In separate analyses for each species, all sequences from homolog clusters containing duplicates were pooled in an all-by-all BLASTn (version 2.2.6, E-value cutoff 1×10^−3^). To classify the level of similarity between duplicates, alignments were manually inspected to identify pairs with no (no hits between duplicates), weak (<200 bp hits that collectively cover less than half of both duplicates), or strong (>80% identity hits over more than half the total length of both members) similarity.

Intron counts for all duplicates are from a search of gene IDs at EuPathDB. Gene-product localization information was harvested from the ApiLoc database [39]. IDs of all duplicates were used to search the ApiLoc database. *C. muris* was the only species without ApiLoc data. Localization data were available for a total of fifty genes, mostly from *P. falciparum* and *T. gondii.*

Comparisons of expression profiles for duplicates identified in *P. falciparum* and *T. gondii* genomes were based on searches of microarray data at PlasmoDB and ToxoDB respectively. For *P. falciparum* strain 3D7, all available time courses of the “Erythrocytic expression time series (3D7,DD2, & HB3)” from [40] were searched based on the expression percentile values. All samples were searched for minimum and maximum expression percentiles of 50 and 100 respectively. For *T. gondii,* all available time courses of the “*T.g.* Cell Cycle (percentile)” from [41] were searched based on the expression percentile values. All samples were searched for minimum and maximum expression percentiles of 50 and 100 respectively. Next we searched for duplicates with similar expression profiles over the same, or shifted, time points for each species. PlasmoDB’s expression profile comparison tool for this data set was used to compare profiles for each gene against the profiles of all other genes. To search over the same time points, a Euclidean search was used that allowed for a maximum 10% of missing data points and a positive correlation with a 0-hour time shift. To search over shifted time points, the same strategy was used, but additional time points were also searched in 6-hour increments (−24, −18, −12, −6, 6, 12, 18, 24 hour time points from 48 total time points). ToxoDB’s expression profile comparison tool for this data set was used to compare profiles for each gene against the profiles of all other genes. To search over the same time points, a Euclidean search was used that allowed for a maximum 10% of missing data points and a positive correlation with a 0-hour time shift. To search over shifted time points, the same strategy was used, but additional time points were also searched in 2-hour increments (−6, −4, −2, 2, 4, 6 time points from 12 time points total). The ‘expression profile comparison’ tools at PlasmoDB and ToxoDB search within an experiment and return genes with similar profiles. There were only a few duplicate pairs with data from each species. Because no statistically significant measure of similarity (e.g. p-value) is returned by this search tool, profile matches between duplicates were manually inspected to verify similarity.

### BLAST2GO Functional Analyses

We installed a local instance of BLAST2GO (B2G) version 2.5.0 [42]. All requisite databases were current at the time of analysis. Protein sequences from all species were run through the B2G pipeline according to documentation instructions. Because of the large number of sequences investigated, BLAST and InterProScan [43] steps were performed independently. All protein-encoding sequences from all species were searched (BLASTp version 2.2.20) against the latest available GenBank nr database with an E-value cutoff of 1×10^−3^. B2G steps used default parameters, with the exception of the HSP cutoff percentage during the annotation step, which was set to 25%. This parameter is intended to guard against spurious domain matches. We tested HSP cutoffs of 50% and 25%. Relaxing this parameter provided annotations for 1,167 additional sequences. InterProScan results greatly enhance the ability of B2G to annotate sequences. InterProScan (version 4.8) was run at the command line for all sequences. The –iprlookup, -goterms, and –nocrc flags were used. The B2G Annex tool was run to augment annotation. To capture as much available functional information as possible, existing GO terms and EC numbers for all genes from EUPathDB were manually incorporated into the B2G data.

To investigate functional trends among putative recently duplicated genes and genes used as markers of segmental duplications, we assembled sets of gene IDs and queried our B2G database for each species. The number of putatively recent duplicate genes with available GO annotations is generally low for all species: *B. bovis* 38/52, *C. muris* 6/12, *C. parvum* 10/12, *N. caninum* 23/36, *P. berghei* 8/26, *P. chabaudi* 4/6 *P. falciparum* 12/12, *P. knowlesi* 11/26, *P. vivax* 32/46, *T. annulata* 6/20, *T. gondii* 59/96, *T. parva* 40/76, for a total of 249/420. The number of genes with GO annotations for markers of segmental duplications is even lower: *B. bovis* 0/96, *P. berghei* 1/24, *P. chabaudi* 7/203, *P. falciparum* 180/216, *T. annulata* 4/19, for a total of 192/558. We also searched the three main GO categories for statistical over- and under-representations of terms. Two-tailed significance tests with an alpha of 0.05 and an FDR correction were used for significance tests. All available protein-encoding genes were used as a reference for each species. Statistical differences were only detected in 5/12 species and were generally weak (data not shown).

## Results

### Terminology

In this study, duplications are defined as protein-encoding paralogs, *i.e.,* at least two copies of a gene within the same genome sequence. Copy number is the total number of paralogs identified in a genome sequence. Multi-copy genes have at least two paralogs. Two-copy paralogs have exactly two copies, and are referred to as duplicates throughout. Tandem duplicates are located near one another on the same chromosome (see Materials and Methods) while dispersed duplicates are on different chromosomes, or distant on the same chromosome. We identified all detectable homologous gene clusters (orthologs and paralogs) in the investigated species (see Materials and Methods). We classified orthologs based on their copy numbers between species. Core genes are defined as having at least one ortholog in all investigated species. Species-specific genes have no orthologs in investigated species. Synteny markers are conserved orthologs in syntenic blocks. Synteny gaps are the non-syntenic regions between syntenic blocks. We define chromosome ends as the areas between the ends of available chromosome sequence and the first syntenic block. Segmental duplications are defined as within-genome synteny detected via paralogous syntenic markers. These regions span multiple genes, but are distinct from whole genome duplications [19].

### Prevalence and Distribution of Duplicates

#### Identification and trends

We used all annotated protein-encoding genes from the twelve species in figure 1 to cluster orthologs and paralogs in all species. Duplicates in each species were identified from the subset of genes with paralogs, regardless of ortholog copy numbers (see Materials and Methods). Gene IDs for all duplicates are in table S1. We classified the chromosomal distribution of all duplicates as dispersed or tandem. Duplications can be created in tandem or as dispersed copies. Tandem gene duplications are generally the result of unequal crossing over that deletes sequence and replaces it with a duplicate copy of a gene. Dispersed gene duplications generally occur through reverse transcription of mRNA intermediates, and insertion of an intronless cDNA into the genome, usually in an unlinked location. Once created, tandem duplications may become dispersed via genome rearrangement. It is less likely that dispersed duplicates will become tandem due to rearrangement. Figure 1 and table 1 contain the numbers of duplicates for each species.

**Table 1:** Duplicate ortholog prevalence and distribution compared to closest relative(s)^a^.

Species	Total #of pairs		# of species-specific pairs		# of pairs withsingle copyortholog		# of pairs withtwo-copyorthologs		# of pairs with>two-copyorthologs	
	D^b^	T^c^	D	T	D	T	D	T	D	T
***P. falciparum***	**26**	**5**	14	3	5	1	7	1	0	0
***P. knowlesi***	**22**	**3**	3	0	12	1	6	2	1	0
***P. vivax***	**29**	**15**	7	5	15	8	6	2	1	0
***P. berghei***	**16**	**9**	1	0	5	8	9	1	1	0
***P. chabaudi***	**19**	**3**	7	2	3	0	9	1	0	0
***B. bovis*** d	**20**	**31**	7	7	10	16	2	1	0	1
***T. annulata***	**7**	**22**	2	1	1	9	3	11	1	1
***T. parva***	**18**	**51**	8	3	3	35	4	11	3	2
***T. gondii***	**59**	**49**	3	8	13	35	42	3	1	3
***N. caninum***	**53**	**19**	3	3	7	11	43	2	0	3
***C. muris***	**24**	**4**	3	0	4	2	17	2	0	0
***C. parvum***	**21**	**7**	1	1	3	3	17	2	0	1

a Closest relative(s) based on Figure 1. Both equally distant relatives were considered for *P. falciparum* and *B. bovis.*

b Pair members are dispersed.

c Pair members are in tandem.

d 7 pairs have different ortholog counts in the equally distant *T. annulata* and *T. parva.*

There are trends in the prevalence and distribution of the 1,062 detected duplicates, both within and among the four lineages (Figure 1). Most duplicates in the genus *Plasmodium* are dispersed. *P. vivax* contains more duplicates (tandem or dispersed) than other *Plasmodium* species, though only 12 more than *P. falciparum*. In contrast, most duplicates within the piroplasms are tandem. Since piroplasms have fewer chromosomes than other species (Figure 2), it is reasonable to expect them to have more pairs on the same chromosome relative to other species, but not necessarily in tandem. *T. annulata* has far fewer duplicates than the other piroplasms, comparable to *Plasmodium*, while *B. bovis* and *T. parva* have roughly double the number. Coccidians have the largest number of duplicates and *T. gondii* has the largest number overall. The numbers of dispersed vs. tandem duplicates are more evenly split in *T. gondii* than in any other species, including *N. caninum,* where most are dispersed. *Cryptosporidium spp*. are the most similar with the same number of duplicates in each species. The majority of *Cryptosporidium* duplicates are dispersed despite high nucleotide similarity.

Circos was used to map the location of duplicates in each genome (Figure 2). Visualization places the data in an intuitive genomic context, where comparisons of genomic characteristics like size and karyotype are easily made, and provides a platform for more detailed comparisons (see below). Most duplicates are localized to chromosomes; with only a few on unassigned contigs (see Materials and Methods). There is a qualitative association of duplicates near chromosome ends in *Plasmodium spp*. and *C. parvum*. The distribution of duplicates is more uniform in *T. gondii* and *T. parva*. These trends hold for other species in each lineage (data not shown), with the exception of *C. muris* where chromosome ends are not available. This is explored quantitatively below. There is a large arc (span across the Circos diagram) connecting *C. parvum* chromosomes three and four in figure 2. These duplicates are ∼25 kb (gene ID cgd3_2180) and ∼40 kb (gene ID cgd4_2900) in size. They represent the very large fatty acid synthase and polyketide synthase respectively. *C. muris, T. gondii*, and *N. caninum* have duplicates in the same ortholog cluster with similar annotations, thus, this duplication is ancient and predates speciation.

#### Copy number evolution

To place the distribution of duplicates in an evolutionary context, we calculated copy numbers of orthologs present in the closest relative(s) for each duplicate pair (Table 1). For equally close relatives, we considered both species (*e.g. T. annulata* and *T. parva* are equally distant from *B. bovis*). Duplicates were classified as species-specific or: single-copy, also two-copy, or greater than two-copy in the closest relative in table 1. Arcs in figure 2 are color-coded accordingly. There are few cases where duplicates have orthologs with a copy number greater than two. These are mostly restricted to the piroplasms and coccidians. All *Plasmodium spp*. have roughly the same number of duplicates that are also two-copy in the closest relative (Table 1). However, other categories show differences within the genus. *P. falciparum* and *P. vivax* have the largest number of species-specific duplicates, while *P. knowlesi, P. vivax,* and *P. berghei* have the largest numbers of duplicates with single-copy orthologs in neighbors. In general, dispersed duplicates outnumber tandem duplicates in all categories for *Plasmodium spp*. As shown in the previous section, *T. annulata* has fewer duplicates than the other piroplasms overall. This is most striking in the species-specific and single-copy categories. In contrast to *Plasmodium spp*., most piroplasm duplicates with single-copy orthologs are in tandem (Table 1). The lack of species-specific and single-copy duplicates in *T. annulata* largely accounts for its overall lack of duplicates compared to other piroplasms, where *B. bovis* and *T. parva* have at least ∼three times as many in each category.

*T. gondii* and *N. caninum* have many duplicates that are either lineage- or species-specific (lineage-specific data not shown). While there are some species-specific *T. gondii* duplicates, the bulk of the difference in prevalence compared to *N. caninum* is in the single-copy category (Table 1). Additionally, the majority of these relative ‘extras’ are in tandem in *T. gondii. C. parvum* and *C. muris*, the basal lineage in this study, share most of their duplicates, and the bulk of them are dispersed. In contrast to other lineages, there is a more uniform distribution for duplicates that are single-copy in the closest relative.

Ortholog copy number differences can be caused by gene gains or losses. Duplicates with two-copy orthologs likely arose prior to speciation. Species-specific duplicates could result from phylum-wide gene losses, or species-specific gains. Duplicates with single-copy orthologs in the closest relative may have been duplicated since speciation, making them putative ‘recent’ duplications, or represent a duplication prior to speciation followed by a loss in one species, making them putative adaptive retentions. Both species-specific and single-copy duplicates may represent adaptations either through gene retention or duplication. With this in mind, the red and green arcs in figure 2 can be viewed as putatively adaptive and/or recent duplications, highlighting, potential genomic ‘hotspots’ for the creation of genes. Further, the trends in prevalence and distribution in table 1 are reinforced in figure 2. For example, *T. gondii* and *T. parva* have many tandem arcs in red, while most *Plasmodium* arcs connect distant loci.

#### Apicomplexan genome architectures

**Figure 3:**
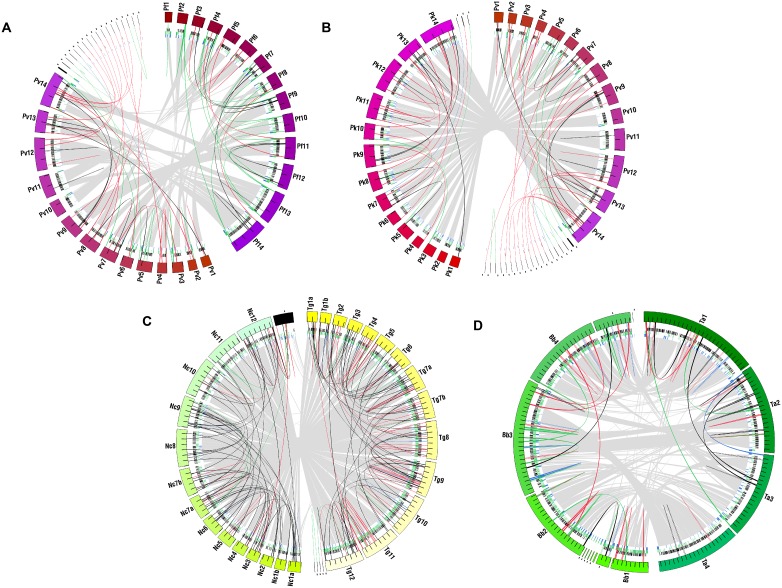
Duplicate genes and with relationship to synteny and genome architecture. Colored gradients on the circles represent genomes as in Figure 2. Unassigned scaffolds/contigs are black. Arcs represent two-copy paralogs as in Figure 2. Gray spans crossing the circle show regions of synteny between genomes. Black, green, and blue ticks on the inner circular tracks between chromosomes and synteny arcs show the locations of core (black), species-specific (green), and multi-copy (blue) genes. Tick marks = 1 Mb in **A,B,C** and 100 kb in **D**. Pf = *Plasmodium falciparum*, Pv = *P. vivax*, Pk = *P. knowlesi*, Tg = *Toxoplasma gondii*, Nc = *Neospora caninum*, Ta = *Theileria annulata*, Bb = *Babesia bovis*.

We used synteny and gene distribution to visualize duplicate distributions in the context of genome architecture evolution. Chromosome ends harbor high numbers of multi-copy and species-specific genes in *Plasmodium spp.*, and to a lesser extent in other lineages [21], [25], [44]. We have observed a similar concentration of repetitive sequences, including multi-copy genes, in syntenic gaps [23] (and data not shown). Synteny between apicomplexan lineages is limited to a few small regions between *Plasmodium* and the piroplasms [23], [26], [45]. We divide non-syntenic regions into chromosome ends (ends), and synteny gaps (gaps) on interior sections of chromosomes. Table 2 contains the numbers of duplicates on ends and in gaps. The boundaries and numbers of syntenic blocks and the relative location of duplicates, depend on the species compared. To ensure that we did not overlook duplicates near gaps, we expanded the search to include 1 kb of sequence adjacent to all ends/gaps. For each comparison, most duplicates are located within syntenic regions and are not on ends or in gaps (Table 2). Selected comparisons are shown in figure 3.

**Table 2:** Number of duplicate genes located in syntenic gaps and on chromosome ends^a^.

Species	# of 2- Copy Genes at Ends^b^	# of 2- Copy Genes in Interior Gaps^b^
***P. falciparum***	33	0
***P. vivax***	18	6
***P. vivax***	18	5
***P. knowlesi***	11	2
***P. chabaudi***	8	1
***P. berghei***	8	1
***P. vivax***	19	7
***P. berghei***	13	2
***P. falciparum***	33	3
***P. berghei***	13	2
***B. bovis***	11	13
***T. annulata***	5	13
***N. caninum***	1	15
***T. gondii***	12	40
***C. parvum***	9	2
***C. muris***	4	1

a Includes genes within 1 kb of either end of a syntenic gap.

b Counts are for individual genes.

Among *Plasmodium spp., P. falciparum* has the largest number of duplicates in ends/gaps for all comparisons, ∼53% (33 genes in gaps/ends divided by 62 total) and ∼58% (36 genes in gaps/ends divided by 62 total) when compared to *P. vivax* and *P. berghei* respectively (Table 2). Other *Plasmodium spp*. have ∼30% of duplicates in ends/gaps, though *P. chabaudi* and *P. berghei* are ∼20% compared to each other. Of these, most are at ends for all species. This is especially striking in *P. falciparum*. Interestingly, all *Plasmodium* duplicates in gaps are either in tandem or on different chromosomes; none are dispersed on the same chromosome (Figure 3 and data not shown). 24/29 total *Plasmodium* duplicates in gaps are either species-specific or single-copy in the closest relative (Calculated from data in Table 2 and Figure 3).

Piroplasms have similar numbers of duplicates in ends/gaps, to one another, and to *Plasmodium spp*. (Table 2). In contrast to *Plasmodium*, piroplasms have a more even split of duplicates in ends and gaps. Most *Babesia* duplicates in gaps are species-specific or single-copy in the closest relative, 11/13. Distribution is split, 8 dispersed vs. 5 tandem (Calculated from data in Table 2 and Figure 3). In *T. annulata* 7/13 are two-copy and 6/13 species-specific or single-copy in the closest relative. In contrast to *Babesia*, most *T. annulata* duplicates in gaps are in tandem, 12/13 (Calculated from data in Table 2 and Figure 3).

Compared to all species, *T. gondii* has the largest number of duplicates in ends/gaps, >3X the number of *N. caninum*. For both coccidians, most are in gaps (Table 2). The majority of *T. gondii* duplicates in gaps are single-copy in *N. caninum,* 26/40, or species-specific, 6/40. Distribution is split with 16/40 dispersed and 24/40 tandem (Calculated from data in Table 2 and Figure 3). 13/15 *N. caninum* duplicates in gaps are dispersed on different chromosomes, and only one is single-copy in *T. gondii* (9 are two-copy in *T. gondii* and 5 are species-specific) (Calculated from data in Table 2 and Figure 3). Comparisons between *Cryptosporidium spp.* are not possible as *C. muris* lacks assembled chromosomes.

There are a few clear qualitative trends in the distribution of duplicates relative to the gene classes shown in figure 3. *Plasmodium* duplicates often appear in areas that contain species-specific genes. In general, they are also found in areas with other multi-copy genes. The most striking trend is the distribution of duplicates that are species-specific or single-copy in the closest relative(s) at chromosome ends. This trend was not detected in the piroplasms. Coccidian duplicates are uniformly distributed along with other gene classes.

### The Fate of Duplicates: Duplicate Divergence

While we cannot ‘see’ duplicates that have met the common fate of removal from reductive genomes, those that were detected may be exploited for innovation. Divergence can take many forms. The nucleotide sequence and resulting protein may be altered resulting in novel function. Relocation and alteration of the surrounding regulatory environment could lead to altered expression. In addition to cases where it is beneficial to retain multiple copies with the same function, duplicates may share an overall role via subfunctionalization, often via different expression patterns. More rarely, novel beneficial functions may emerge via neofunctionalization [7]. By examining the modes of divergence we can learn about the biological roles of duplicates.

#### Sequence analysis

Similarity at the amino acid level was established during homolog clustering (see Materials and Methods). However, this does not guarantee a ‘high’ degree of nucleotide similarity between duplicates. We measured nucleotide similarity in the representatives *P. falciparum* and *T. gondii*. Only **∼**15% of *P. falciparum* and **∼**23% of *T. gondii* duplicate pairs are strongly similar to each other (see Materials and Methods, data not shown) indicating that they are either rapidly diverging or older duplications. Degree of nucleotide similarity does not appear correlated with distribution in either species. A trend observed in both species is that duplicates with strong similarity, amino acid or nucleotide, tend to have fewer orthologs (data not shown). For less similar duplicates with orthologs there was often a clear division in the cluster, with each member of a duplicate pair more similar to orthologs within and between genera than their paralog, consistent with duplication and divergence prior to speciation. Taken together, this points towards the long-standing role of duplicates as a means of innovation in apicomplexans.

#### Intron counts

Differential intron counts for duplicate gene pairs can indicate divergence and imply the mechanism of duplication. If one member of a duplicate pair has no introns, this is indicative of duplication via reverse transcription of a processed mRNA intermediate. We compared intron counts for all duplicates and correlated them with distribution (Table 3). Across all lineages, the distribution of duplicates in each category of intron counts in Table 3 recapitulates overall distribution trends from figure 1. Duplicates with different intron counts are most abundant in the coccidians and *T. parva*, and generally underrepresented in other lineages. Duplicates with one intron-less member were counted separately. They are most common in *Plasmodium spp*. where they are usually dispersed. Most *Cryptosporidium* duplicates have no introns. This is unsurprising as introns are largely absent in the genus, a common feature of genome reduction [46].

**Table 3:** Comparison of duplicate gene intron numbers.

Species	# of Pairs with Same Intron Count^a^		# of Pairs with Different Intron Count^a^		# of Pairs with No Introns		# of Pairs with One Intron-less Member	
	D^b^	T^c^	D	T	D	T	D	T
***P. falciparum***	10	3	4	0	6	2	6	0
***P. knowlesi***	7	0	4	1	5	2	6	0
***P. vivax***	4	6	2	4	11	1	12	4
***P. berghei***	5	0	3	1	4	4	4	4
***P. chabaudi***	9	1	2	0	4	2	4	0
***B. bovis***	6	11	3	5	8	12	3	3
***T. annulata***	1	7	2	5	4	10	0	0
***T. parva***	4	10	4	19	7	16	3	6
***T. gondii***	17	8	34	24	6	11	2	6
***N. caninum***	12	8	32	2	5	5	4	4
***C. muris***	0	0	0	0	18	4	6	0
***C. parvum***	0	0	0	0	21	7	0	0

a Both members have > = 1 intron.

b Pair members are dispersed.

c Pair members are in tandem.

#### Gene expression

Duplicate genes can diverge via changes in their promoters (or changes of their promoters via relocation) that permit temporal and abundance changes in gene expression. We searched available expression data to explore similarities and differences in temporal gene expression profiles among duplicates (see Materials and Methods). We focused on *P. falciparum* and *T. gondii* because of the availability of relatively extensive expression data for these species. Duplicates with expression data for each paralog were limited (Figure 1 and Table 4). Gene expression levels measured across multiple time points form a ‘profile’, with peak expression at one measured time point. Genes with similar profiles, over the same time points, have highly similar expression profiles. Genes may also have similar profiles that are shifted in time, with peak expression earlier or later. These may also be considered similar profiles, though ‘shifted’. To differentiate between these, expression profiles were searched with and without time-shifts. *P. falciparum* has six duplicate pairs, mostly dispersed, with similar profiles (with and without time-shifts) and six pairs, also mostly dispersed, with different profiles (Table 4). The majorities of pairs with data are dispersed for both species. For *T. gondii,* the high number of pairs that are both dispersed and have different expression profiles, points toward a correlation between separation of duplicates in the genome and diverging expression, though data are limited. We observed no trends in intron count, ortholog copy number, or sequence similarity among those with different expression profiles (data not shown). This is not entirely unexpected as most are dispersed, making altered promoters a likely cause of expression differences.

**Table 4:** Comparison of duplicate gene expression profiles.

Species	# of Pairs with Microarray Data		# of Pairs with Similar Profiles		# of Pairs with Similar Time-Shifted Profiles		# of Pairs with Different Profiles	
	D^a^	T^b^	D	T	D	T	D	T
***P. falciparum***	9	3	1	2	3	0	5	1
***T. gondii***	24	9	6	2	0	1	18	6

a Pair members are dispersed.

b Pair members are in tandem.

#### Subcellular localization

One of the most easily observed indicators of divergence between paralogs is the differential subcellular localization of gene products. We searched for experimentally verified evidence of apicomplexan protein localization in the ApiLoc database (see Materials and Methods). We found nine cases where each duplicate product has localization evidence (Figure 1 and Table S2). In each case, the duplicate genes are dispersed on different chromosomes. Eight of the duplicates share the same, or similar, functional annotation. The remaining duplicate has one member annotated as hypothetical. EuPathDB gene IDs, annotated functions, and subcellular locations of gene products are in table S2. Most (7/9) duplicates have differences in the localization of their gene products. In the remaining cases, a clear comparison could not be made. In two of the duplicate pairs, *C. parvum* polyketide synthases and *T. gondii* acetyl co-a carboxylases it appears that one copy of the pair was acquired by horizontal transfer from a prokaryote and intracellular gene transfer from an endosymbiont, respectively, rather than via duplication [47]–[51].

#### Functions of recently duplicated genes

Are there identifiable functional trends in putative recent duplicates (single-copy in the closest relative(s))? More than half of apicomplexan genes are annotated with unknown or hypothetical functions (Table 5). This severely limits the analysis of gene functions. To directly tackle this problem and provide as much functional information as possible, we used BLAST2GO (B2G) to augment functional information (see Materials and Methods). Combining B2G annotations with available GO terms from EuPathDB, we more than doubled the number of sequences with GO terms (Table 5). As expected given the limited data, attempts to detect significant enrichments or deficits of GO terms for each species were unsuccessful (see Materials and Methods).

**Table 5:** BLAST2GO and EuPathDB annotation results.

**Total Sequences**	60455
**Annotated as ‘Hypothetical’ or ‘Function Unknown’**	33020 (54.6%)
**Sequences With GO Terms Before BLAST2GO**	16938 (28%)
**Sequences With GO Terms After BLAST2GO**	32703 (54.1%)
**Sequences With GO Terms After EuPathDB Incorporation**	34979 (57.9%)

We examined the most-specific Molecular Function GO terms for each species in figure S1 to determine trends within and between species. As expected, few terms are shared between species. Many, including shared terms, are too broad for meaningful interpretation. For example: DNA binding, Protein binding, DNA-directed RNA polymerase activity, and others. Protease activity terms were found in all lineages. Proteases are key players in all apicomplexan lifecycle stages, including many aspects of the host-pathogen interaction such as immune evasion and, tissue invasion [52], [53]. We identified six types of peptidase activities among recent duplicates: amino- in *P. falciparum* and *C. parvum,* cysteine- in *P. berghei*, *T. parva*, and *N. caninum*, serine- in *P. chabaudi*, *B. bovis*, and *N. caninum*, aspartic- in *B. bovis*, metallo- in *C. muris*, and unspecified in *T. gondii* (figure S1).

Innovation can also be provided by changes in copy number, rather than changes in function. We identified a known pair of *T. gondii* tyrosine hydroxylases (tyrosine 3-monooxygenase) as possible recent duplicates [54]. Our analyses showed that these genes, TGME49_012740 and TGME49_087510, have a single apicomplexan ortholog in *N. caninum.* They are highly similar, have the same number of introns and are on the same chromosome, separated by 58 genes over ∼450 kb. Further investigation at OrthoMCL DB [55] confirmed their copy number in the Apicomplexa and identified orthologs in animals, protozoa, bacteria, and algae (see Discussion). TGME49_012740 has been shown to increase dopamine metabolism via production of the rate-limiting step in dopamine synthesis [56]. Together, these findings are suggestive of a potential link between the function of a recently duplicated gene and parasite-induced alteration of host behavior.

### Segmental Duplications Appear Rare

#### Identification and trends

**Figure 4:**
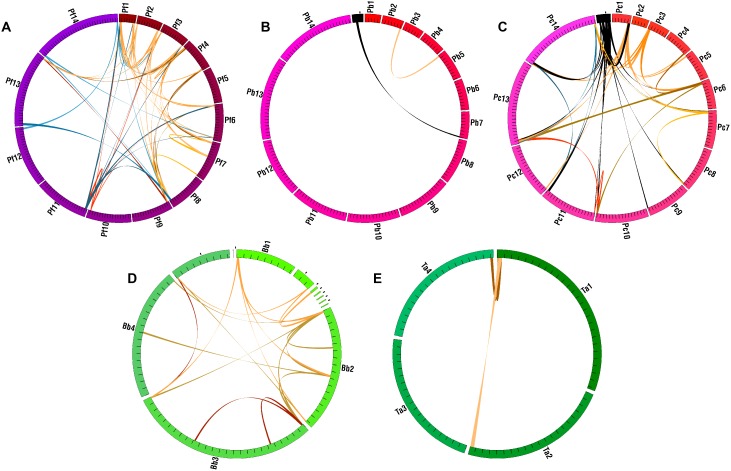
Distribution of segmental duplications. Colored gradients on the circles represent genomes as in Figure 2. Unassigned scaffolds/contigs are black. Spans show segmental duplications in five species (**A–E**). Black spans have at least one end on an unassigned contig. Other colors are arbitrary and delineate spans. Pf = *Plasmodium falciparum*, Pb = *P. berghei*, Pc = *P. chabaudi*, Bb = *Babesia bovis*, Ta = *Theileria annulata.*

Homologs identified by OrthoMCL were used to detect putative segmental duplications as regions of ‘paralogous synteny’ via MCSCAN (see Materials and Methods). Gene IDs for all markers in segmental duplications are in table S3. The most striking aspect of this analysis is the overall dearth of segmental duplications in the Apicomplexa. They were found in only five of twelve species, with 2–57 duplicated segments in each genome (Figure 1). The lack of segmental duplications appears to be another feature of reductive apicomplexan genomes. Nearly all segmental duplications are concentrated on chromosome ends (Figure 4). It is possible that this dearth may be an artifact of assembly errors (See Discussion).

#### Functional enrichment contained in segmental duplicates

Functional analyses were limited by the lack of genes with annotations, even after B2G enhancement (see Materials and Methods). B2G was used to compare the functions of segmental duplication marker genes against all annotations for each species and to look for significant over or under represented functions. Only *P. falciparum* and *T. annulata* had detectably significant differences. Genes on *T. annulata* segmental duplicates (4 total) are enriched for ATP binding and ATPase activity coupled to transmembrane movement (data not shown). Genes on *P. falciparum* segmental duplicates (180 total) are significantly lacking in ATP binding and ATPase activity categories. Significant functional enrichments in the main GO categories indicate roles in pathogenesis and antigenic variation. Enriched categories include: host cell plasma membrane, infected host cell surface knob, Maurer’s cleft, antigenic variation, pathogenesis, rosetting, host cell surface binding, and cytoadherance to microvasculature (figure S2). Enrichments in these GO terms are somewhat expected since the genes are located on chromosome ends where genes implicated in infection and pathogenesis are localized. Less expected are enrichments for several mitochondrial processes: cytochrome c oxidase activity, mitochondrial electron transport, and respiratory chain complex (figure S2).

## Discussion

### 

#### Duplications shape genomic landscapes

Broadly speaking, any novel genomic feature that escapes removal is an ‘innovation’, though our ability to identify functional innovations is limited by our understanding of gene functions and organism biology. This is especially true in reductive genomes, where gene loss predominates and genomic real estate is at a premium. This feature of apicomplexan genome architecture is highlighted by the lack of segmental duplications and two-copy genes with more than two copies in closest relative(s) (Figure 1 and Table 1). The processes of gene duplication and maintenance are among the forces that shape genomic landscapes. By mapping detectable duplications (genes and genomic segments) we learn which regions of the genome, in duplicate, are tolerated by the organisms (at least thus far), and where, within the genomic landscapes duplications occur. Further, hypotheses about the timing of duplications are possible based on their distribution among species and sequence divergence. General timing of duplications, combined with knowledge of host-pathogen interactions, provides a functional context for the exploration of the functional consequences of duplications. This is especially exciting and challenging given the evolutionary time frame involved. The Apicomplexa are an ancient phylum that diverged ∼420 million years ago (mya) [29], [30]. Comparisons at this scale can be likened to those made across the entire chordate lineage [57].

Apicomplexan genomes are dynamic and prone to extensive rearrangement relative to other examined phyla [23]. Previously, genomic repeats (genes or other repeats) have been treated as a single genomic feature used only to highlight areas of genomic interest. Here we have taken the next step in unraveling the relationship between duplications and genome architecture with a systematic focus on their distribution across the phylum. Tandem duplicates are somewhat rare and are likely either under selection to remain proximal, or recent enough to have not yet been dispersed. It is possible that some tandem duplications may remain undetected due to assembly errors (see below). In coccidians and piroplasms, duplicates with single-copy orthologs in the closest relative(s) are mostly tandem (Table 1). This finding correlates with the hypothesis that these duplications occurred after the species diverged. This trend is not observed in *Plasmodium* or *Cryptosporidium spp.* where most duplicates are dispersed, regardless of copy number in the closest relative(s). Estimates place the radiation of *Plasmodium spp.* ∼130–150 (mya) [58], [59] and the last common ancestor of *T. gondii* and *N. caninum* at ∼28 mya [60]. If one accepts these divergence times, then it appears that *T. gondii* has accumulated duplicates faster than *Plasmodium spp.* Further, *T. gondii*, with the largest overall genome size and largest number of duplicates, also has the widest host range of the investigated species.

Duplicated genes are one class of genomic repeats. Repetitive sequences facilitate genomic rearrangements via homologous recombination. We observe varying concentrations of duplicates in and around interior syntenic gaps in coccidians and piroplasms (Table 2). This suggests a correlation between duplicated sequence and genome rearrangement. Is duplication restricted to these genomic locations? Are these ‘hotspots’ of gene creation? Interestingly, most *N. caninum* duplicates in syntenic gaps are also two-copy in *T. gondii.* However, the reverse is not true. Most *T. gondii* duplicates in syntenic gaps are single-copy in *N caninum (* Figure 3 and data not shown). It is attractive to think that these duplicates have been generated since these species diverged and that their genomic location is evidence of the relationship between duplication and genome architecture. Similarly, 24/29 *Plasmodium* duplicates in syntenic gaps are either single-copy in the closest relative(s) or species-specific. Syntenic gaps may be ‘hot spots’ for gene creation across the phylum. However, in contrast, to coccidians and piroplasms, the majority of *Plasmodium* duplicates in non-syntenic regions are located on dynamic chromosome ends. While the specific chromosomal region (syntenic gap, chromosome end, or both) differs with lineage, duplications are often associated with and concentrated in dynamic genomic regions.

### Duplicate Diversification Tracks Evolutionary Trajectory

Genome sequences are palimpsests that reveal their evolutionary history [20]. Documentation of the modes of duplicate divergence informs on the forces that drive genomic innovation. Comparison of gene sequences and intron counts provide a gross look at duplicate divergence and mechanism of duplication. 10/31 *P. falciparum* and 66/108 *T. gondii* duplicates have no detectable nucleotide similarity. Most of these duplicates are dispersed in each species: 80% in *P. falciparum* and 55% in *T. gondii*. Duplicates with nucleotide similarity, weak or strong (See Materials and Methods), are also mostly dispersed, 18/21 (16 weak, 5 strong) in *P. falciparum* and 23/42 (17 weak, 25 strong) in *T. gondii.* In nearly every measure of nucleotide similarity and distribution, dispersed duplicates outnumber tandem duplicates. The exception is highly similar *T. gondii* duplicates, where tandem duplicates outnumber dispersed 15 vs. 10. It is possible that highly similar, tandem duplicates may have been missed due to collapsed genome assemblies (see below). Duplicate genes with one intron-less member are in the minority in all species. It follows that duplication via reverse transcription of an RNA intermediate is likely not a primary mechanism of gene duplication. This is not surprising considering the dearth of transposable elements, including retrotransposons, observed thus far, though telomere-associated reverse transcriptase domains are present in several species (data not shown). Trends vary across lineages, but once again, *T. gondii* breaks ranks with the largest number of duplicates with different intron counts (Table 3). It is intriguing to hypothesize that the relatively wide host range of *T. gondii* may be facilitated by the increased divergence of duplicated genes.

Duplicate genes that are maintained may reveal genes that are beneficial for the parasite. Duplicate genes with products involved in environmental interaction, hosts in this case, are more likely to be maintained [18]. Trends and patterns that highlight this are unfortunately difficult to detect given that the majority of apicomplexan gene functions remain ‘in the dark’. Despite these limits we have provided at least a partial light to allow a better view of the functions of duplicates (figure S1). Proteases are critical to pathogenicity and host interaction [52], [53], [61]. They are also among recent duplicates found in 8/12 species, despite roughly 40% of genes having no GO terms. While this is not entirely unexpected given that many genes in each species that have protease or protease-annotated annotations, the broad categories of different proteases identified in the functions of recent duplicates reinforce their importance.

A pair of *T. gondii* tyrosine hydroxylase genes appear directly related to host pathogen interaction, and alteration of host behavior [54], [56]. A putative mechanism for such alterations was recently proposed [62]. Interestingly, they are differentially expressed; one in the bradyzoite stage and the other constitutively [54]. To better understand their evolution, we queried OrthoMCL DB [55]. Both genes belong to the same OrthoMCL DB cluster. At the time of analysis, of the 45 genes in the cluster, there were: 26 metazoan (including humans, other primates, and rodents), 5 plant (1 moss, 2 green algae, 1 red algae, and 1 diatom), 4 kinetoplastid, 3 apicomplexan (2 *T. gondii* and 1 *N. caninum*), 2 *Tetrahymena thermophile* (an Alveolate, along with apicomplexans), 3 unclassified eukaryote, and 2 bacterial genes. A BLASTp search of the GenBank (BLAST version 2.2.29, search performed 12/2013) confirmed the OrthoMCL DB range and also detected similarity to a gene from *Perkinsus marinus* a dinoflagellate, even closer to the Apicomplexa. Could a gene have been acquired in the last common ancestor of *N. caninum* and *T. gondii* and duplicated in *T. gondii?* Acquisition seems more parsimonious than loss in all other apicomplexans, but the reductive nature of their genomes cannot be ignored and a loss in all other examined apicomplexan lineages is a possibility.

### Limits of Detection

The number of duplications (both genes and larger genome segments) detected in this study likely underrepresents the biological reality. As with any pan-genomic analysis, the constraints of available genome assemblies and annotation apply. We are particularly concerned about collapses of identical or nearly identical genome segments into a single sequence segment as has been documented in several assemblies [63]–[68]. As available data (especially long-read data) and assembly methods improve, the pool of duplicate regions available for analysis will grow. Two potential sources of error should be understood and both stem from the difficulty inherent in proving biological absence. First, ortholog clustering is heavily dependent on the species included, their assemblies and annotations, and parameter selection. Missing orthologs and paralogs will affect the results. We have dealt with this where possible. Likewise, ‘collapsed’ assemblies could hinder the detection of duplicate genes and segmental duplications. For example, this is a possible culprit behind the lack of duplicates in *T. annulata* compared to *T. parva* (Figure 1). Further, if significant portions of a biological genome are represented by unassigned contigs the detection of segmental duplications will be hindered, especially if they contain only a few genes each. This is particularly true for *T. gondii* and *P. vivax*, with 381 and 2,763 unassigned contigs respectively. All totals should be considered as a baseline. Through careful selection of genome data, parameter optimization, and curation of results (see Materials and Methods) we sought to minimize false positives and negatives. Second, some duplicate genes may be the result of one copy arriving via horizontal or intracellular gene transfer (HGT or IGT). We found two such cases in the literature (Table S2). Targeted phylogenetic analysis is required to rule out HGT or IGT.

### Conclusions and Future Directions

We have taken a first step towards a better understanding of apicomplexan gene and segmental duplication, divergence, and how duplications relate to overall biology. This systematic investigation lays the groundwork for further work with emerging data on gene function and new genome sequences from other apicomplexan species. We have identified clear trends and interesting differences in the prevalence, distribution, divergence, and biological innovation achieved in apicomplexan species and lineages (Figure 1). These are guideposts to those studying both genome evolution and parasite biology. While care must be taken with conclusions made from the current data, many of these trends are consistent with the patterns emerging from studies of apicomplexan genome evolution. Apicomplexan genomes are reductive in nature and highly dynamic with respect to genome rearrangement where form serves function and where the ‘typical’ mode and tempo observed in model eukaryotes does not necessarily apply. Repeats, duplicated genes or other genomic features, can facilitate genome rearrangements by serving as recombination sites. Rearrangements do not exist in a ‘vacuum’ and genes and/or regulatory elements can be relocated, causing changes in gene expression [69]. Duplication of genes and genomic segments is correlated with overall genome architecture, and we show that their divergence and exploitation is likely a significant component in the continuum of forces that have helped shape these genomes.

## Supporting Information

Figure S1 **Most specific molecular function GO terms for putative recent duplicates by species.** Pie charts show the number of genes with detected function from BLAST2GO analyses. Not all genes identified as putative recent duplicates were associated with functions.
(PDF)

Figure S2 **Differential GO term distribution and most specific molecular function GO terms for** ***P. falciparum*** **segmental duplication markers.** Differential GO term distribution analysis was performed with BLAST2GO. The ‘Test Set’ is from markers in segmental duplications. The ‘Reference Set’ is all apicomplexan genes with GO terms. All differences are significant (see Materials and Methods). Enrichments were calculated across all three GO categories. The pie chart shows the number of genes with detected function from BLAST2GO analyses.
(PDF)

Table S1 Gene IDs for detected two-copy paralogs.
(TXT)

Table S2 Subcellular localization of duplicates.
(XLSX)

Table S3 Gene IDs for detected segmental duplications.
(TXT)
