# Supplementary material for: A Survey of Innovation through Duplication in the Reduced Genomes of Twelve Parasites
Source: PLoS One. 2014 Jun 11;9(6):e99213. doi: 10.1371/journal.pone.0099213 (PMC4053351; doi:10.1371/journal.pone.0099213)
Supplement: Figure S1 — Most specific molecular function GO terms for putative recent duplicates by species. Pie charts show the number of genes with detected function from BLAST2GO analyses. Not all genes identified as putative recent duplicates were associated with functions. (PDF) [file pone.0099213.s001.pdf]

Supplemental Figure 1

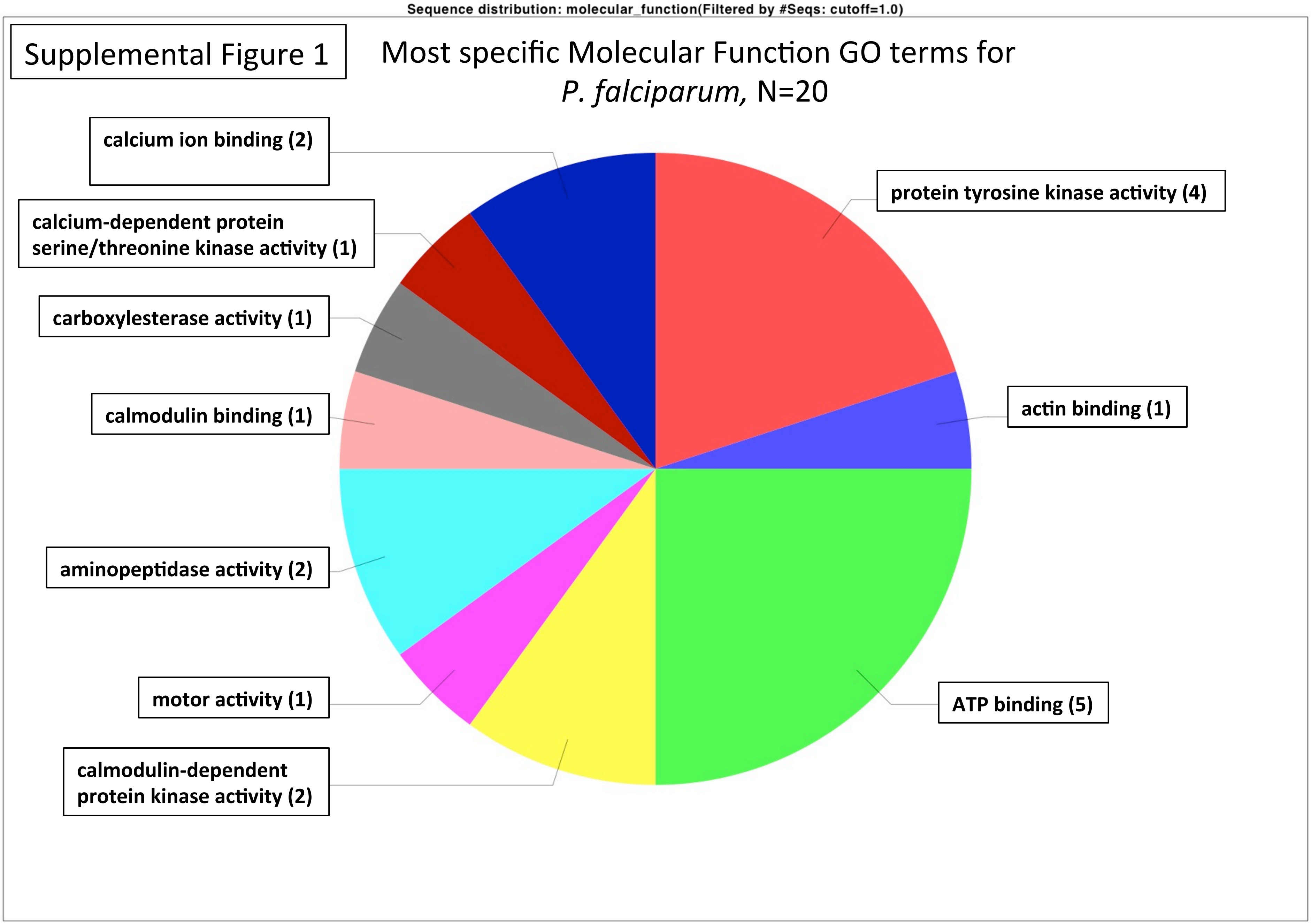

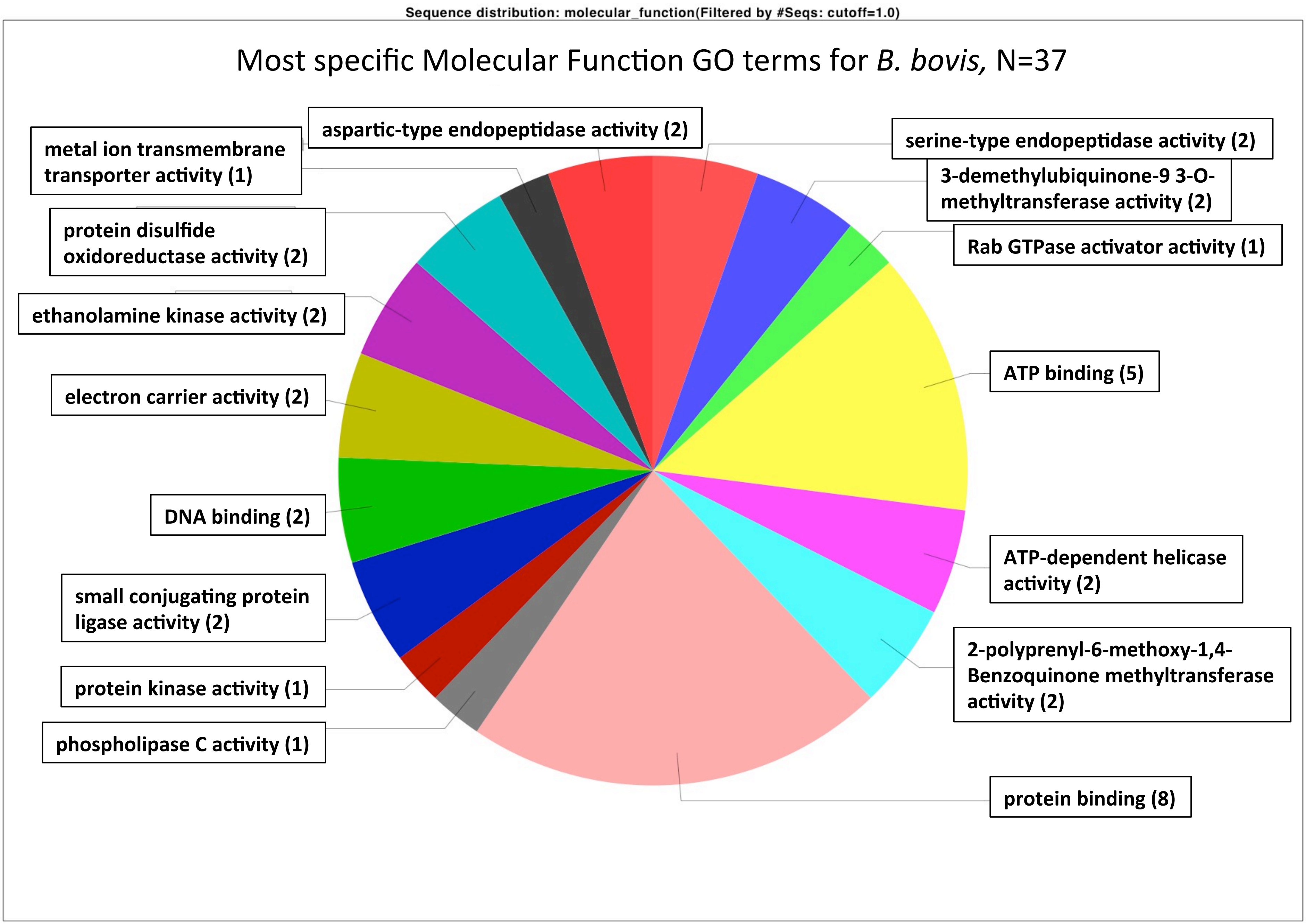
Sequence distribution: molecular_function(Filtered by #Seqs: cutoff=1.0)
Most specific Molecular Function GO terms for B. bovis, N=37
aspartic-type endopeptidase activity (2)
metal ion transmembrane transporter activity (1)
protein disulfide oxidoreductase activity (2)
ethanolamine kinase activity (2)
electron carrier activity (2)
DNA binding (2)
small conjugating protein ligase activity (2)
protein kinase activity (1)
phospholipase C activity (1)
serine-type endopeptidase activity (2)
3-demethylubiquinone-9 3-O-methyltransferase activity (2)
Rab GTPase activator activity (1)
ATP binding (5)
ATP-dependent helicase activity (2)
2-polyprenyl-6-methoxy-1,4-Benzoquinone methyltransferase activity (2)
protein binding (8)

Most specific Molecular Function GO terms for *C. muris,* N=14

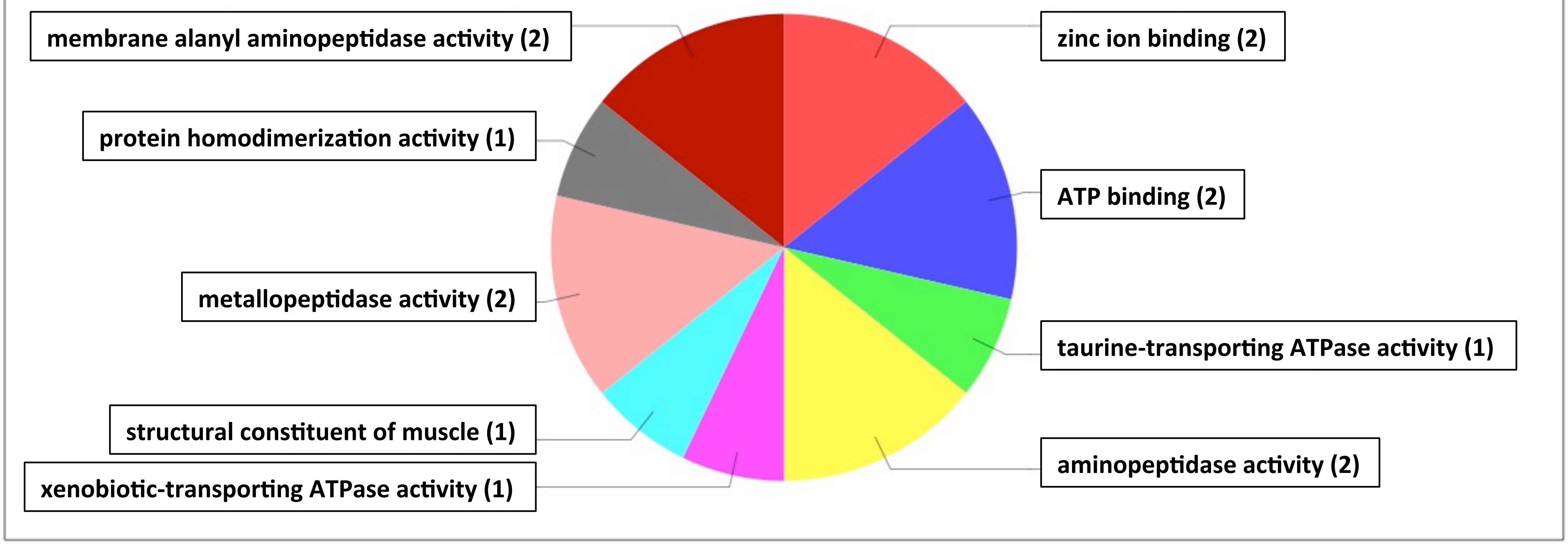

Sequence distribution: molecular_function(Filtered by #Seqs: cutoff=1.0)

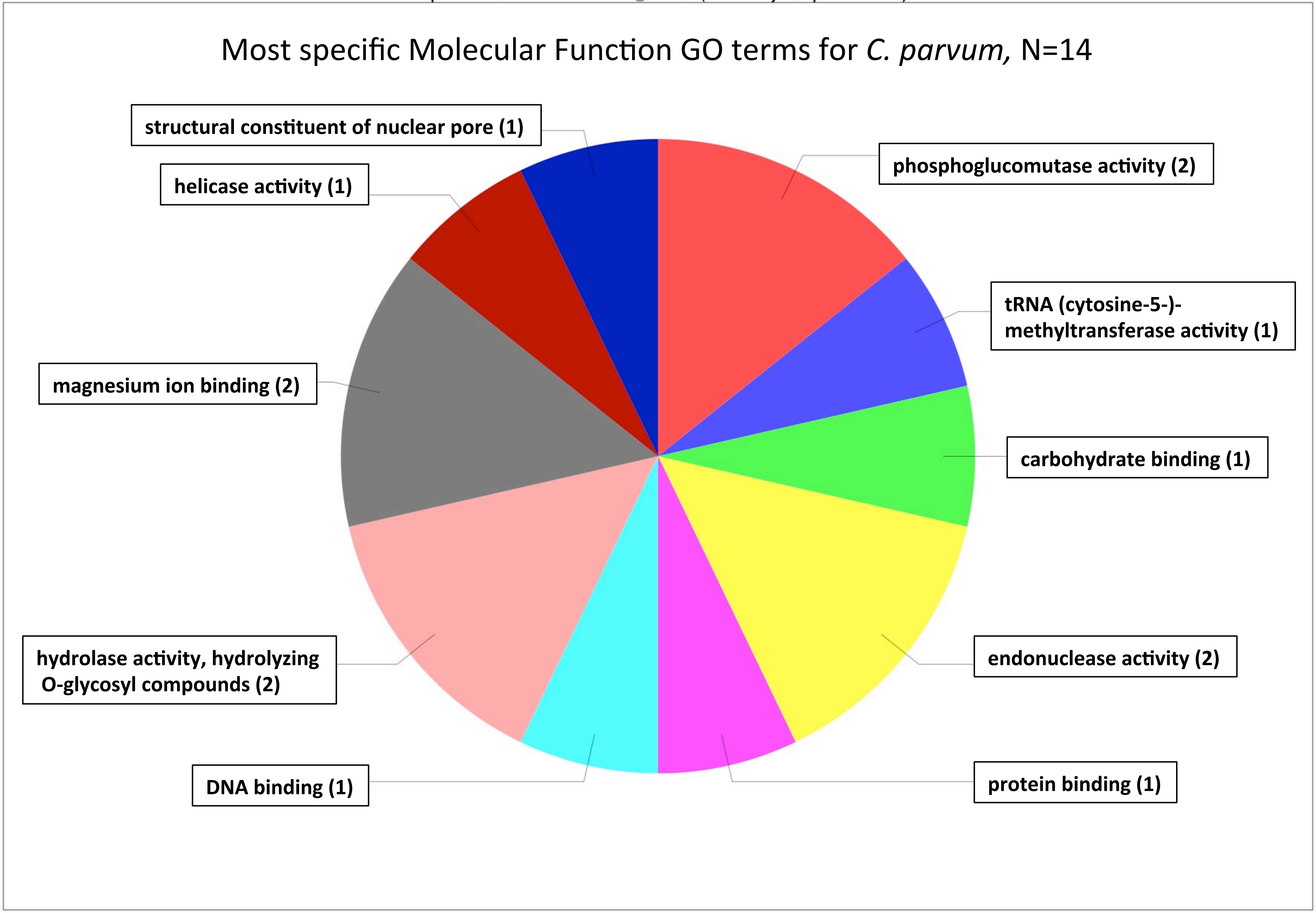

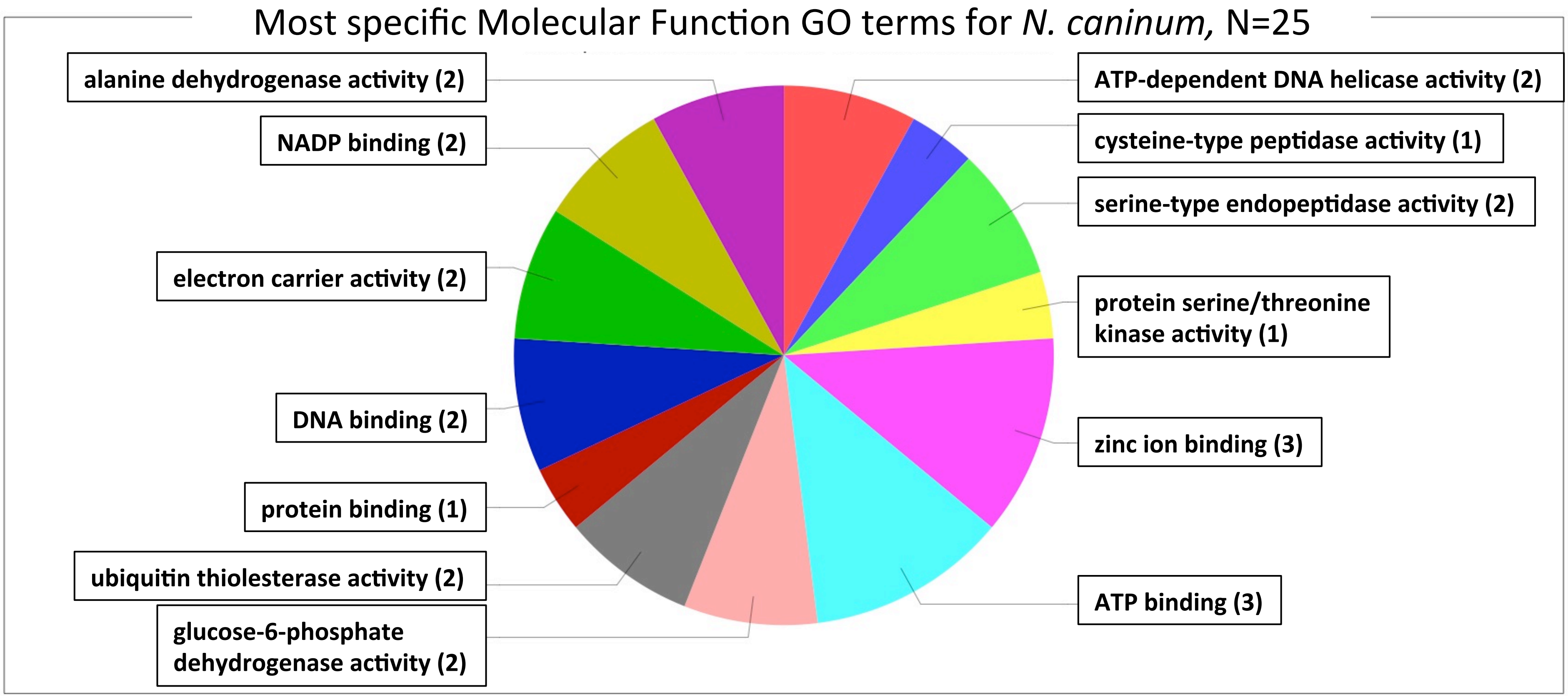

Most specific Molecular Function GO terms for *N. caninum*, N=25
alanine dehydrogenase activity (2)
NADP binding (2)
electron carrier activity (2)
DNA binding (2)
protein binding (1)
ubiquitin thiolesterase activity (2)
glucose-6-phosphate dehydrogenase activity (2)
ATP-dependent DNA helicase activity (2)
cysteine-type peptidase activity (1)
serine-type endopeptidase activity (2)
protein serine/threonine kinase activity (1)
zinc ion binding (3)
ATP binding (3)

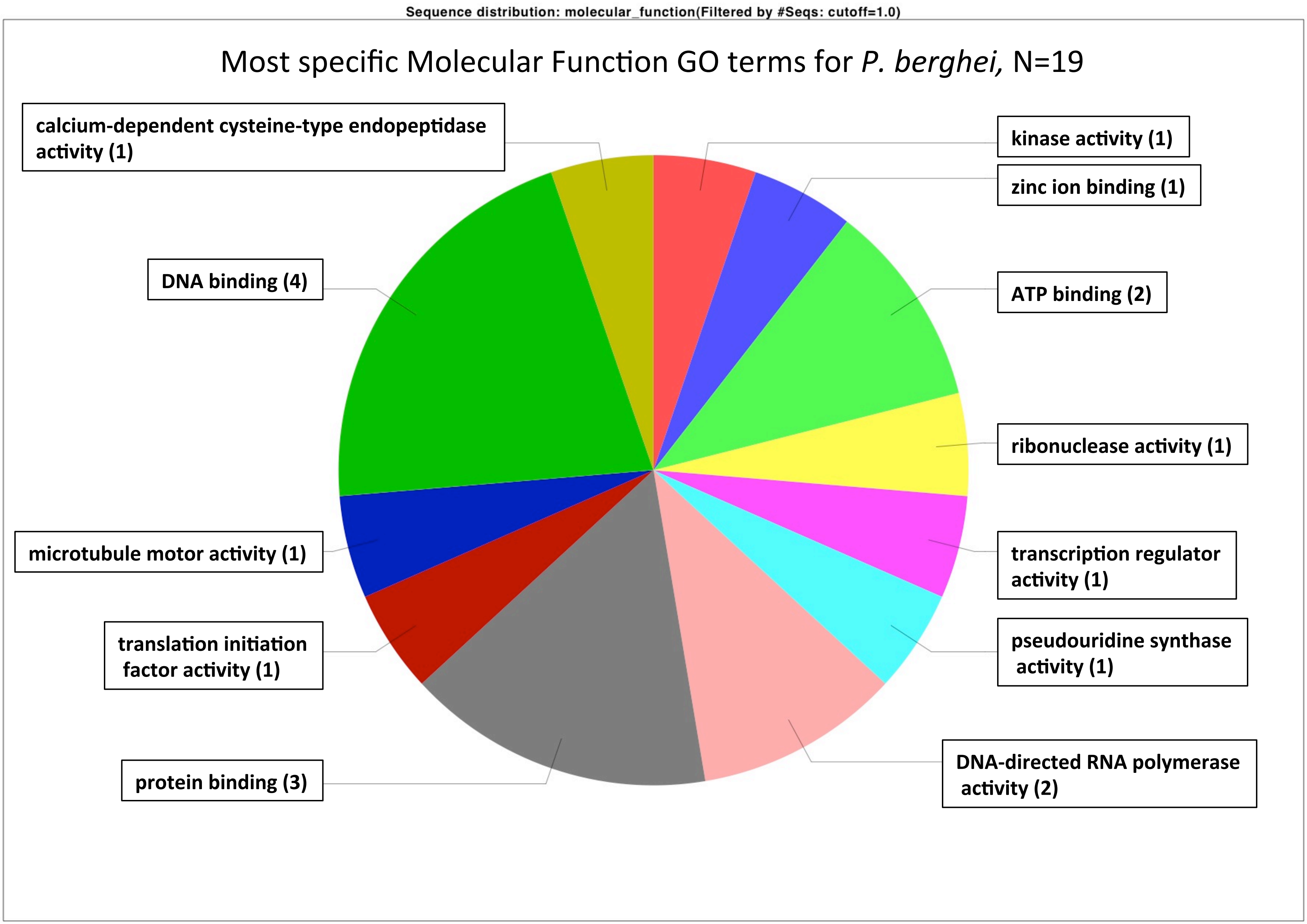
Sequence distribution: molecular_function(Filtered by #Seqs: cutoff=1.0)
Most specific Molecular Function GO terms for P. berghei, N=19
calcium-dependent cysteine-type endopeptidase activity (1)
kinase activity (1)
zinc ion binding (1)
DNA binding (4)
ATP binding (2)
ribonuclease activity (1)
microtubule motor activity (1)
transcription regulator activity (1)
translation initiation factor activity (1)
pseudouridine synthase activity (1)
protein binding (3)
DNA-directed RNA polymerase activity (2)

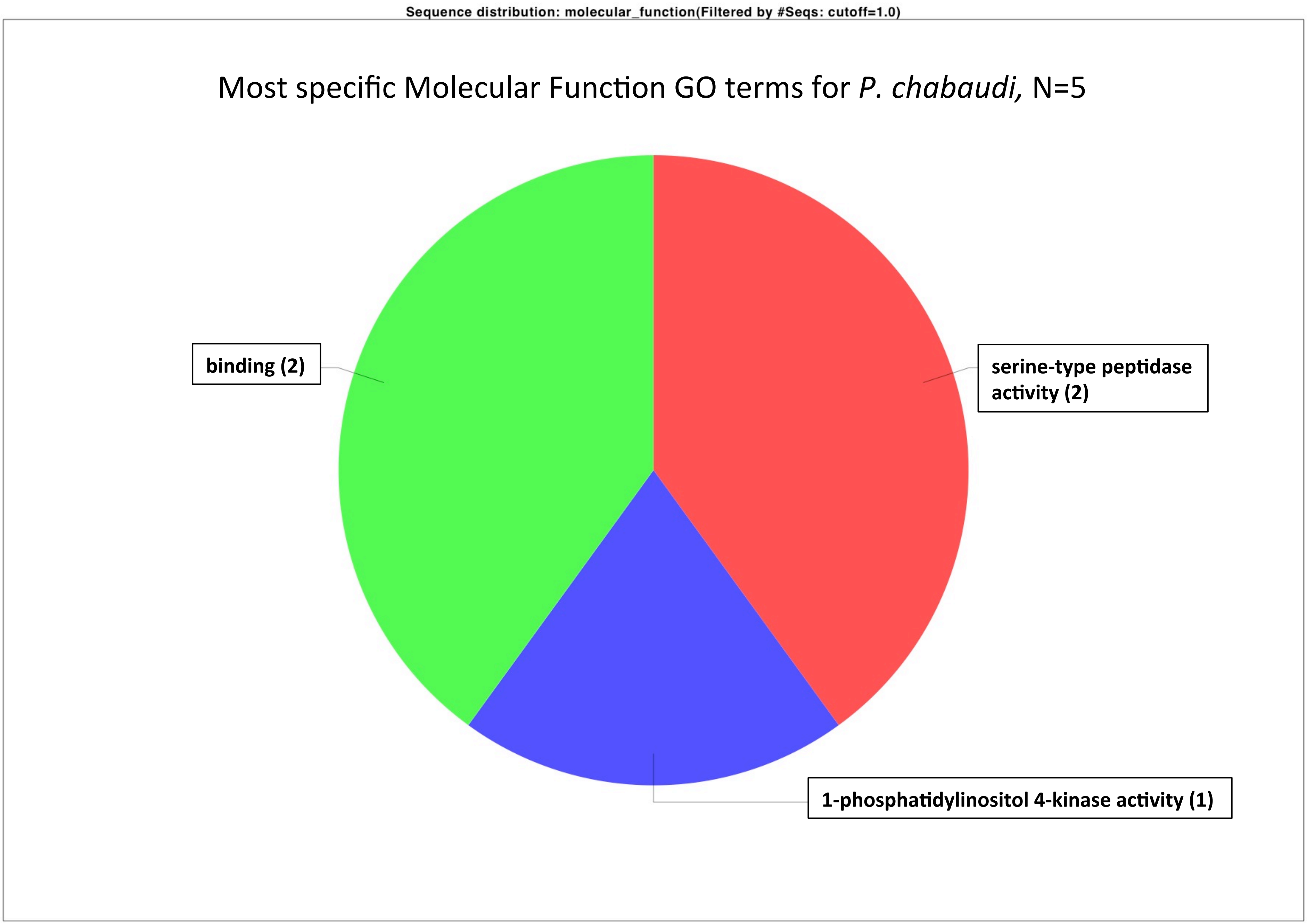

Sequence distribution: molecular_function(Filtered by #Seqs: cutoff=1.0)
Most specific Molecular Function GO terms for *P. chabaudi,* N=5
binding (2)
serine-type peptidase activity (2)
1-phosphatidylinositol 4-kinase activity (1)

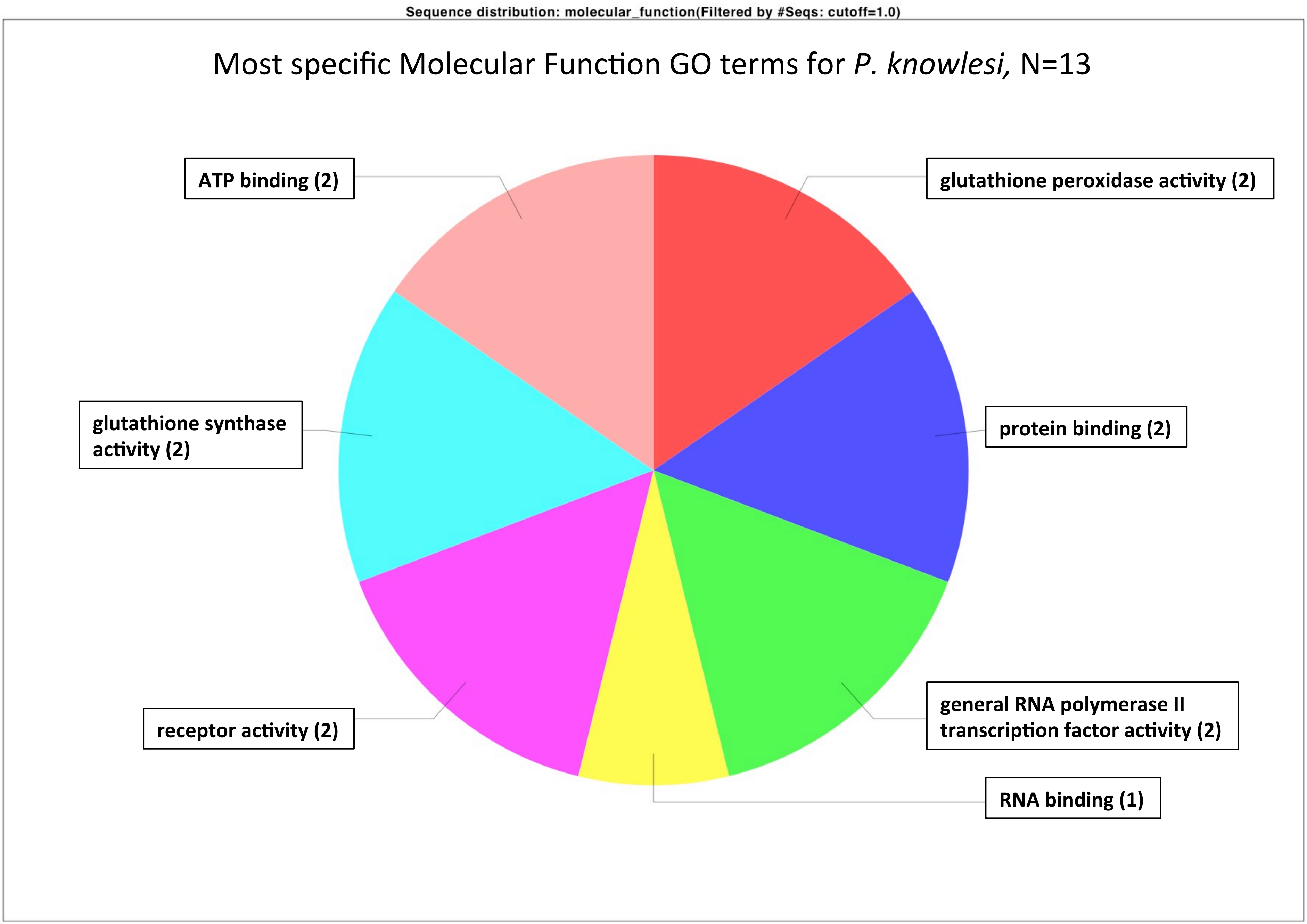
Sequence distribution: molecular_function(Filtered by #Seqs: cutoff=1.0)
Most specific Molecular Function GO terms for P. knowlesi, N=13
ATP binding (2)
glutathione peroxidase activity (2)
glutathione synthase activity (2)
protein binding (2)
receptor activity (2)
general RNA polymerase II transcription factor activity (2)
RNA binding (1)

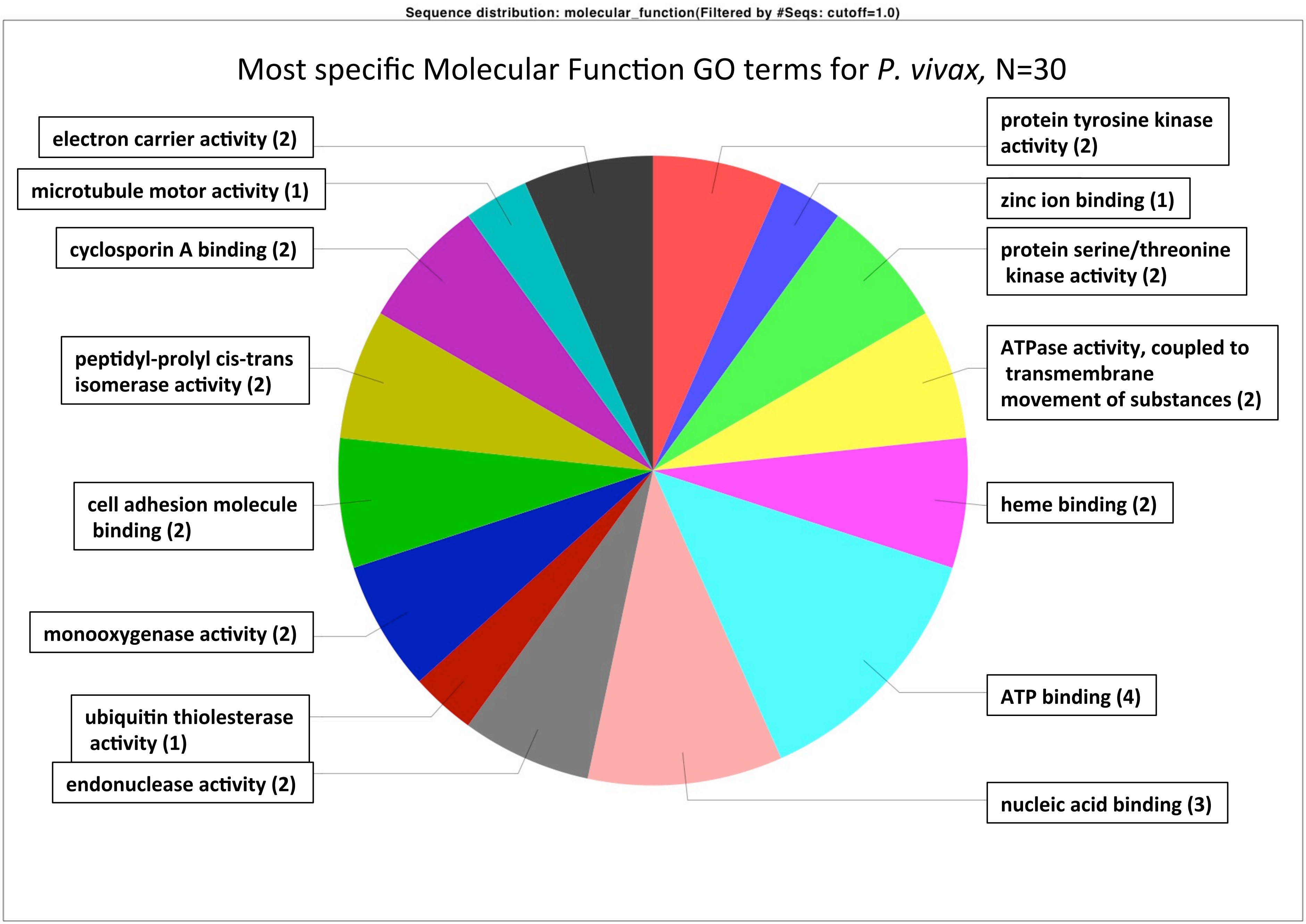
Sequence distribution: molecular_function(Filtered by #Seqs: cutoff=1.0)
Most specific Molecular Function GO terms for P. vivax, N=30
electron carrier activity (2)
microtubule motor activity (1)
cyclosporin A binding (2)
peptidyl-prolyl cis-trans isomerase activity (2)
cell adhesion molecule binding (2)
monooxygenase activity (2)
ubiquitin thiolesterase activity (1)
endonuclease activity (2)
protein tyrosine kinase activity (2)
zinc ion binding (1)
protein serine/threonine kinase activity (2)
ATPase activity, coupled to transmembrane movement of substances (2)
heme binding (2)
ATP binding (4)
nucleic acid binding (3)

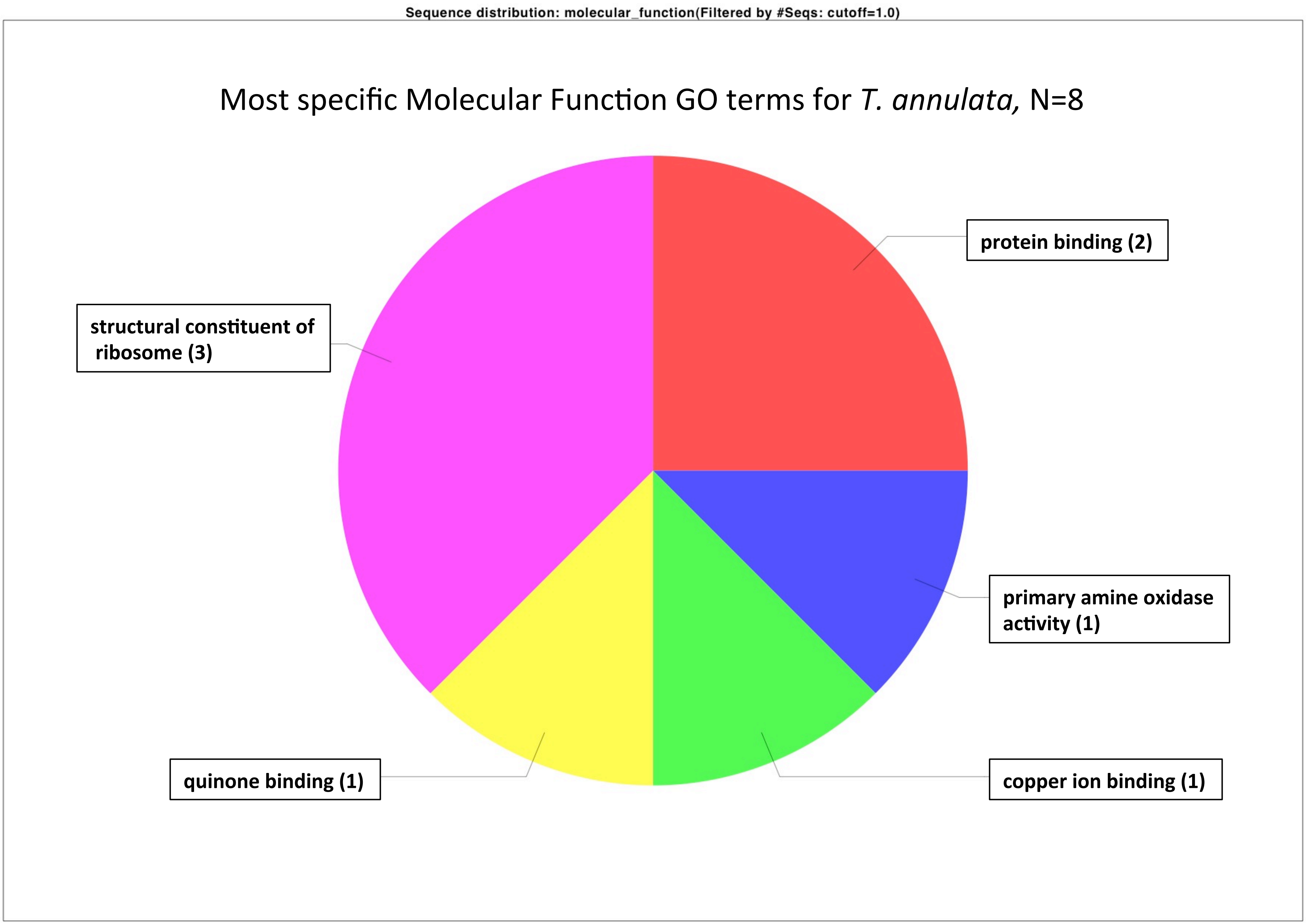
Sequence distribution: molecular_function(Filtered by #Seqs: cutoff=1.0)
Most specific Molecular Function GO terms for T. annulata, N=8
protein binding (2)
structural constituent of ribosome (3)
primary amine oxidase activity (1)
quinone binding (1)
copper ion binding (1)

Sequence distribution: molecular_function(Filtered by #Seqs: cutoff=1.0)

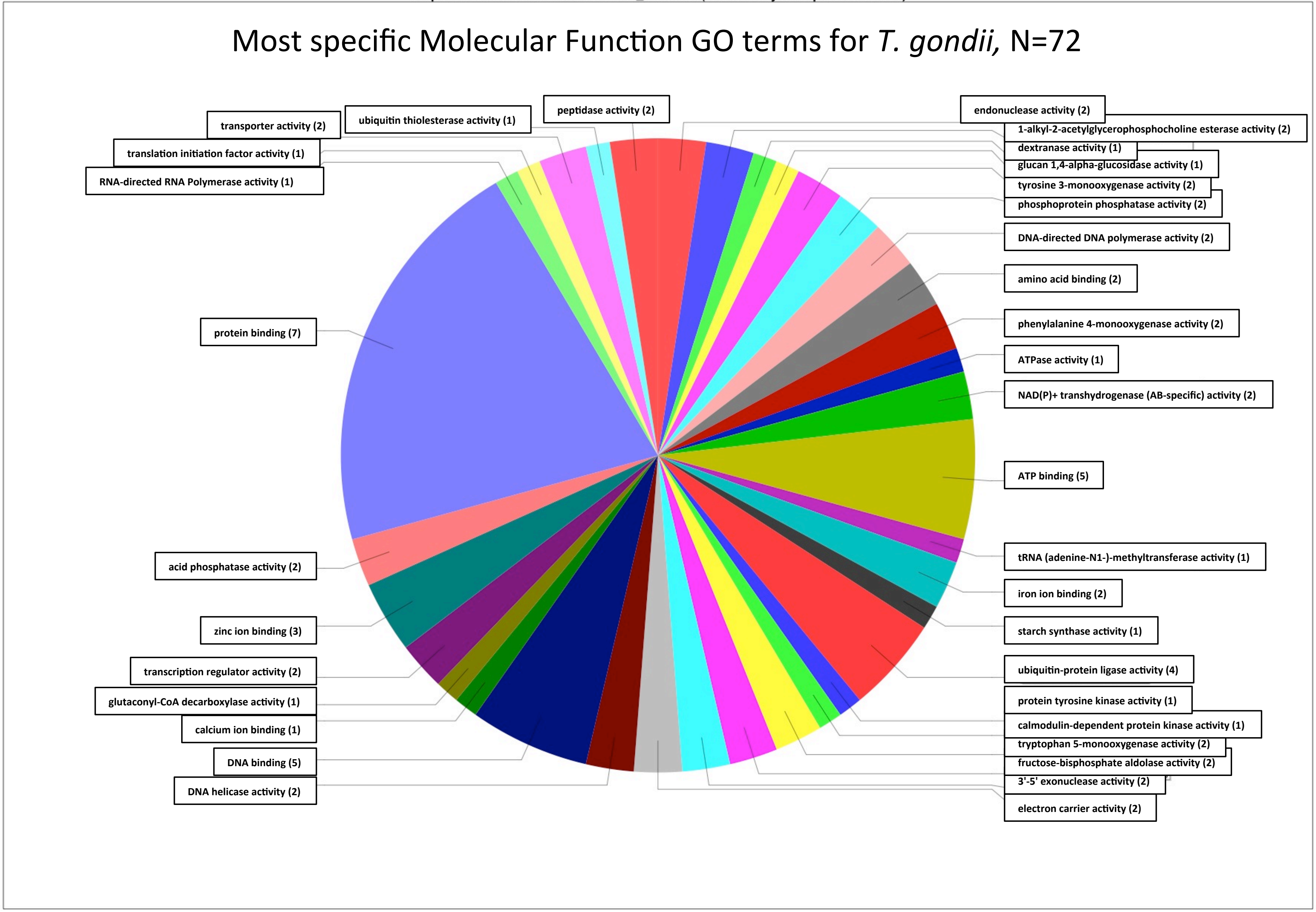

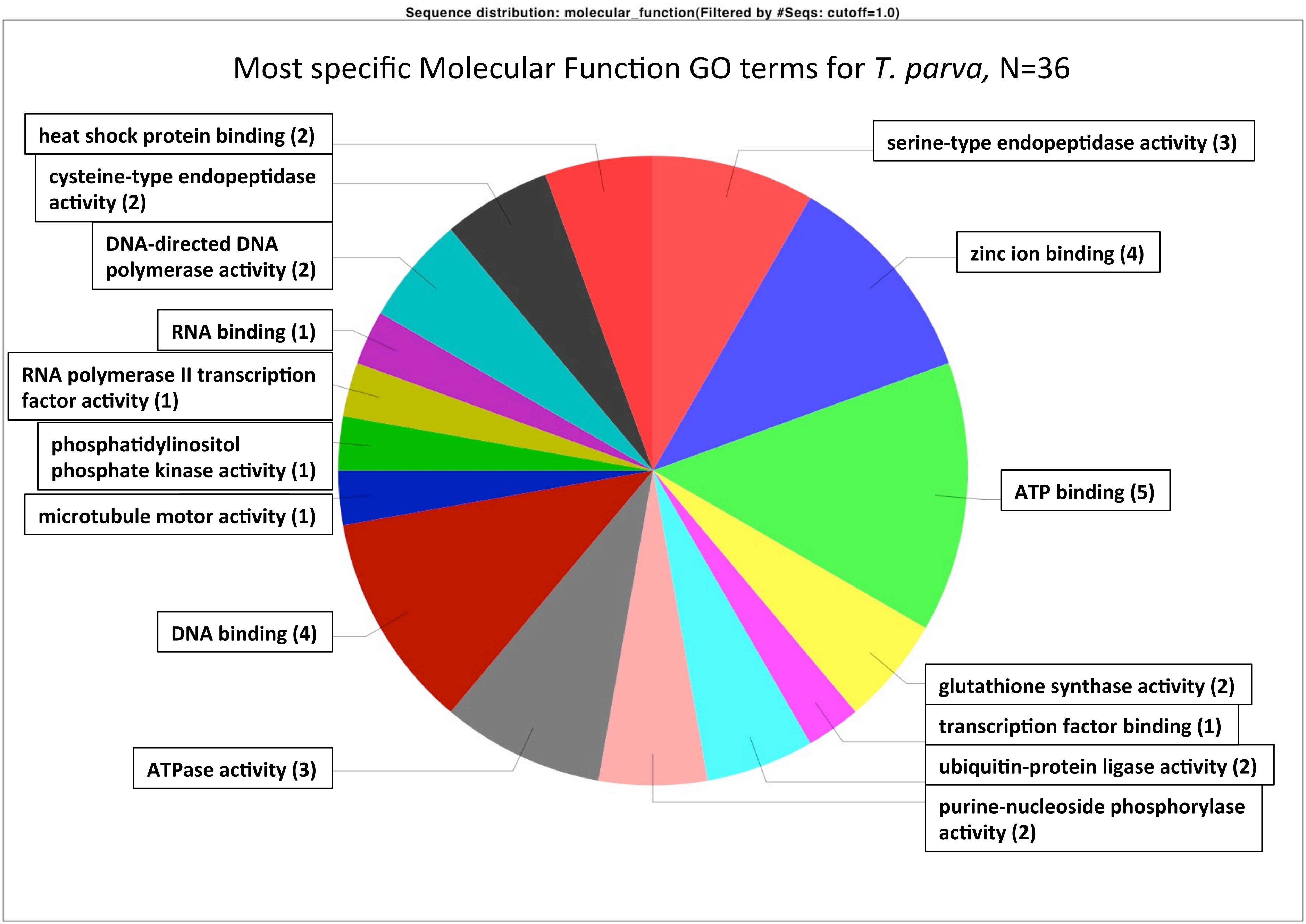
Sequence distribution: molecular_function(Filtered by #Seqs: cutoff=1.0)
Most specific Molecular Function GO terms for T. parva, N=36
heat shock protein binding (2)
cysteine-type endopeptidase activity (2)
DNA-directed DNA polymerase activity (2)
RNA binding (1)
RNA polymerase II transcription factor activity (1)
phosphatidylinositol phosphate kinase activity (1)
microtubule motor activity (1)
DNA binding (4)
ATPase activity (3)
serine-type endopeptidase activity (3)
zinc ion binding (4)
ATP binding (5)
glutathione synthase activity (2)
transcription factor binding (1)
ubiquitin-protein ligase activity (2)
purine-nucleoside phosphorylase activity (2)
