# Supplementary material for: A Survey of Innovation through Duplication in the Reduced Genomes of Twelve Parasites
Source: PLoS One. 2014 Jun 11;9(6):e99213. doi: 10.1371/journal.pone.0099213 (PMC4053351; doi:10.1371/journal.pone.0099213)

Supplemental Figure 2

P. falciparum segmental duplication markers

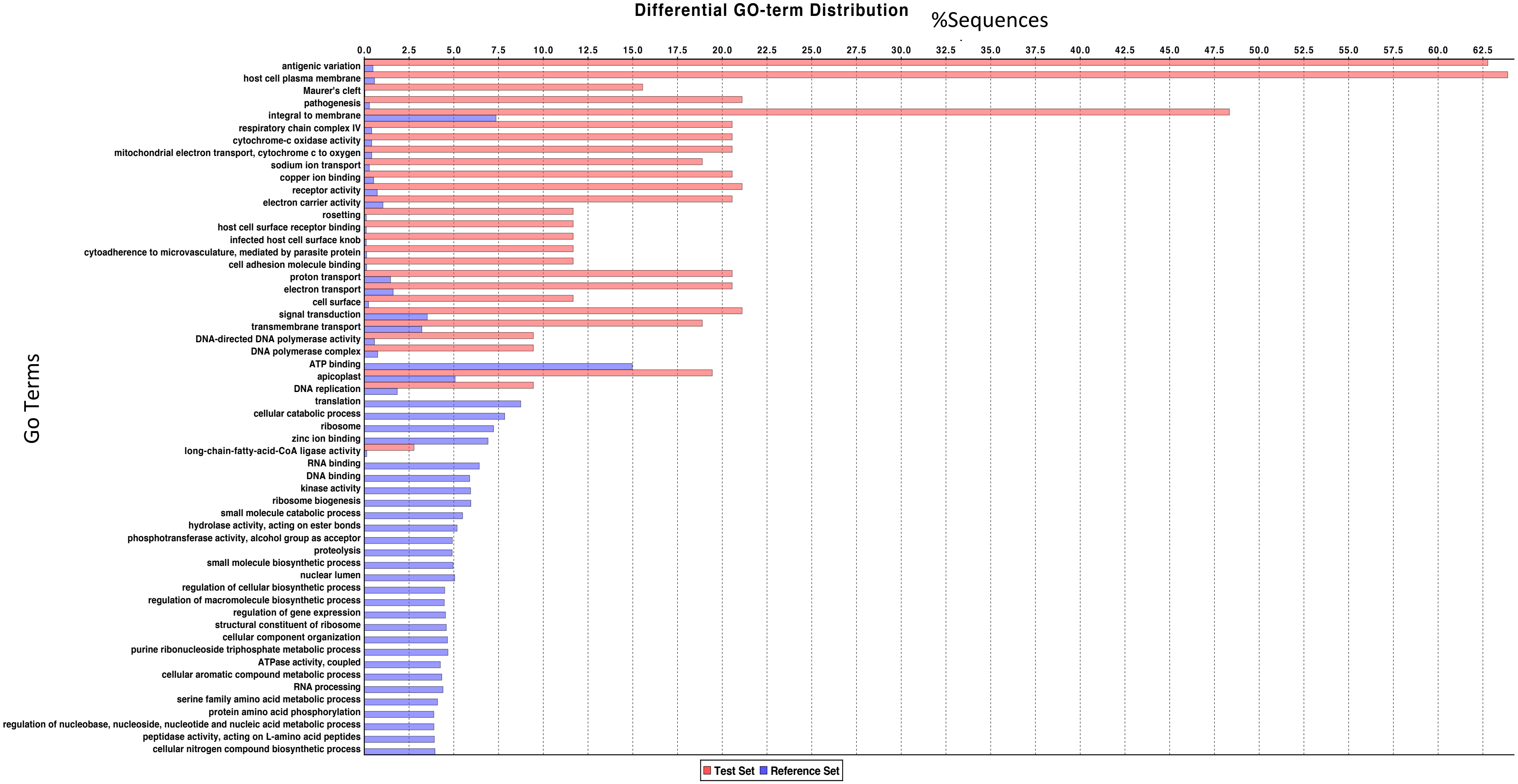

Most specific Molecular Function GO terms for *P. falciparum*, N=191

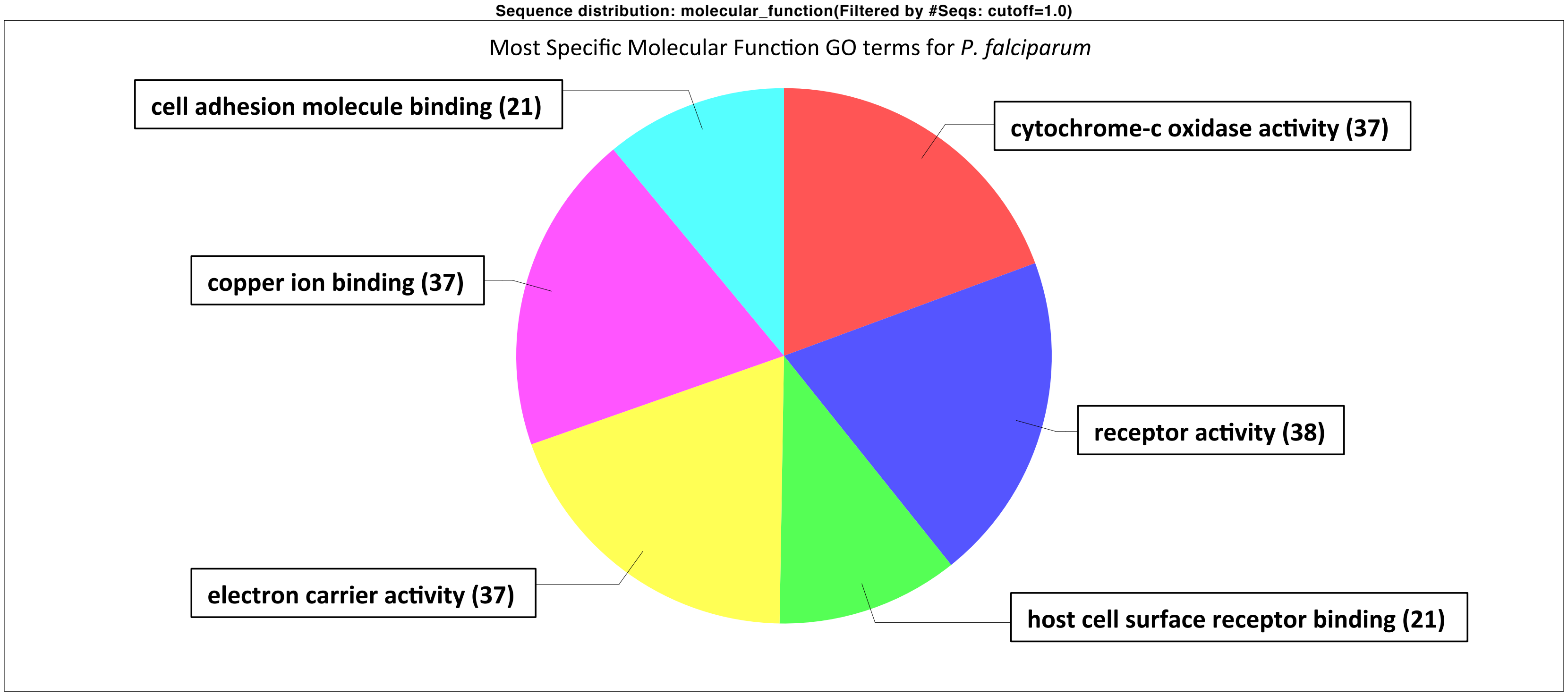

Supplement: Figure S2 — Differential GO term distribution and most specific molecular function GO terms for P. falciparum segmental duplication markers. Differential GO term distribution analysis was performed with BLAST2GO. The ‘Test Set’ is from markers in segmental duplications. The ‘Reference Set’ is all apicomplexan genes with GO terms. All differences are significant (see Materials and Methods). Enrichments were calculated across all three GO categories. The pie chart shows the number of genes with detected function from BLAST2GO analyses. (PDF) [file pone.0099213.s002.pdf]
